# Mechanisms and applications of natural plant ingredients in modulating amino acid metabolism for the improvement of diabetic retinopathy: a review

**DOI:** 10.3389/fendo.2026.1745507

**Published:** 2026-02-12

**Authors:** Le-yi Zhang, Tian-yi Zhang, Ya-juan Zheng, Jia-xin Li, Hu-rong Chen, Jing Cao, Teng Dan

**Affiliations:** 1 He University, Shenyang, China; 2 He Eye Specialist Hospital, Shenyang, China; 3 Dalian Medical University, Dalian, China; 4 Liaoning University of Traditional Chinese Medicine, Liaoning, China

**Keywords:** amino acid metabolism, application research, diabetic retinopathy, mechanisms, natural drugs

## Abstract

Diabetic Retinopathy (DR) is a leading cause of vision loss in diabetic patients, driven by oxidative stress, inflammation, and vascular abnormalities. Recent studies highlight amino acid metabolism abnormalities, particularly in glutamate, arginine, and tryptophan, as critical factors in DR pathogenesis. Preclinical evidence suggests that these metabolic disturbances may contribute to retinal neurodegeneration and vascular damage, offering potential new targets for therapy. Natural plant-derived compounds, such as flavonoids, catechins, and alkaloids, have been shown in animal and cell culture studies to regulate amino acid metabolism and may offer therapeutic potential for DR, although clinical validation remains limited. These compounds exhibit antioxidant, anti-inflammatory, and neuroprotective properties. Flavonoids improve amino acid accumulation, reduce oxidative stress, and protect retinal cells, while catechins enhance amino acid synthesis and redox balance. Alkaloids like berberine regulate nitric oxide synthesis, improving retinal microcirculation and endothelial function. In DR, glutamate excess activates NMDA receptors, leading to retinal neuronal toxicity, while arginine and tryptophan metabolism abnormalities further disrupt vascular and immune function. Natural drugs targeting these pathways could alleviate oxidative stress, inflammation, and retinal damage. However, challenges remain in clinical application, including low bioavailability and variability in product quality. Future research should focus on multi-omics integration, personalized medicine, and clinical trials to establish robust evidence for the use of natural drugs in DR treatment, ultimately improving long-term visual outcomes and quality of life.

## 1 Introduction

Diabetic retinopathy (DR) is the leading cause of vision loss and blindness among working-age adults globally, affecting approximately 35% of patients with diabetes. The worldwide prevalence of DR is projected to reach 191 million cases by 2030, imposing a substantial economic and social burden on healthcare systems (1). The pathogenesis of DR involves complex and interconnected pathological processes, including oxidative stress, chronic inflammation, vascular endothelial dysfunction, and retinal neurodegeneration. Persistent hyperglycemia triggers a cascade of metabolic disturbances, leading to increased generation of reactive oxygen species (ROS), activation of inflammatory signaling pathways, and progressive damage to retinal microvasculature and neural cells (2–4).

Current therapeutic strategies for DR primarily focus on glycemic control, anti-vascular endothelial growth factor (anti-VEGF) therapy, and laser photocoagulation. Although these interventions can delay disease progression, significant limitations remain: anti-VEGF treatment requires frequent intravitreal injections with associated risks of endophthalmitis and retinal detachment; laser therapy often results in permanent visual field defects; and optimal glycemic control alone cannot completely prevent the onset or progression of DR (5, 6). Moreover, these approaches mainly target late-stage vascular complications rather than addressing the underlying metabolic dysfunction that initiates and sustains retinal damage. Therefore, there is an urgent need for novel therapeutic strategies that can target early pathogenic mechanisms, provide neuroprotection, and offer improved long-term efficacy and safety profiles.

In recent years, amino acid metabolism has emerged as a critical mechanistic link in the pathophysiology of DR, offering novel therapeutic targets. Metabolomic profiling studies have revealed significant alterations in key amino acid pathways in patients with DR, particularly those involving glutamate, arginine, tryptophan, and branched-chain amino acids (BCAAs) (7, 8). Emerging evidence suggests that these metabolic abnormalities may not be merely secondary consequences of hyperglycemia but appear to actively contribute to retinal neuronal damage and vascular dysfunction through multiple mechanisms: preclinical studies indicate that glutamate excitotoxicity may induce neuronal apoptosis via excessive activation of N-methyl-D-aspartate (NMDA) receptors; dysregulated arginine metabolism disrupts nitric oxide (NO) homeostasis, impairing retinal blood flow and endothelial function; activation of the tryptophan-kynurenine pathway promotes immune dysregulation and chronic inflammation; and BCAA accumulation triggers inflammatory responses through mechanistic target of rapamycin complex 1 (mTORC1) signaling in retinal Müller cells (9, 10). Understanding these amino acid metabolic disturbances provides novel mechanistic insights into DR pathogenesis and identifies potential intervention points for therapeutic development.

Natural plant-derived compounds have emerged as promising therapeutic candidates for modulating amino acid metabolism in DR due to their multi-target, multi-pathway mechanisms of action and generally favorable safety profiles. Unlike conventional single-target pharmaceuticals, natural compounds such as polyphenols (flavonoids, catechins, resveratrol), alkaloids (berberine), and terpenoids can simultaneously address multiple pathological processes including oxidative stress(summarized in **Table 1**), inflammation, and metabolic dysregulation (27, 28). For example, Salvia miltiorrhiza, traditionally used for cardiovascular disease management, may benefit DR patients through its antioxidant, anti-inflammatory, and lipid metabolism-regulating properties (29). Resveratrol has demonstrated the ability to modulate oxidative stress, suppress inflammatory pathways, and provide neuroprotection in preclinical DR models (30). Importantly, emerging evidence suggests that these natural compounds may specifically target amino acid metabolic pathways: polyphenols can modulate glutamate homeostasis and enhance antioxidant enzyme activity; berberine influences arginine-NO metabolism; and terpenoids may regulate enzymes in the tryptophan-kynurenine pathway (31, 32). However, clinical translation of these compounds faces significant challenges, including low bioavailability, product quality variability, limited large-scale human trials, and incomplete mechanistic understanding, necessitating systematic evaluation of the current evidence base.

**Table 1:** Summary of oxidative stress and inflammatory mechanisms.

Mechanism type	Key molecules/pathways	Major pathological effects	References
ROS Generation	Mitochondrial electron transport chain, NADPH oxidase	Lipid peroxidation, DNA damage, protein oxidative modification	(11, 12)
Polyol Pathway	Aldose reductase, sorbitol	Cellular osmotic imbalance, redox state disturbance	(13)
PKC Activation	PKC-β, PKC-δ	Increased vascular permeability, hemodynamic changes	(14, 15)
AGEs Formation	AGEs-RAGE axis	Inflammation amplification, apoptosis, vascular dysfunction	(16, 17)
Pro-inflammatory Factors	TNF-α, IL-6, IL-1β	Apoptosis, leukocyte adhesion, vascular leakage	(18–20)
NF-κB Pathway	IKK complex, p65/p50	Pro-inflammatory gene transcription, chronic inflammation	(21, 22)
MAPK Pathway	ERK, JNK, p38	Cellular stress response, pro-inflammatory factor production	(2, 23)
Inflammasome	NLRP3, Caspase-1	IL-1β and IL-18 maturation, pyroptosis	(20, 24)
Epigenetic Regulation	DNA methylation, histone modifications	Long-term gene expression changes, metabolic memory	(25, 26)

This systematic review aims to achieve three primary objectives:

Characterize amino acid metabolic abnormalities in DR pathogenesis: Synthesize evidence regarding specific alterations in glutamate, arginine, tryptophan, and BCAA metabolism in DR, evaluating the quality and consistency of findings from human and animal studies. Critically assess the mechanistic links between these metabolic disturbances and key pathological features of DR, including oxidative stress, inflammation, neurodegeneration, and vascular dysfunction. Identify knowledge gaps regarding causal relationships, temporal dynamics, and stage-specific metabolic profiles.

Evaluate natural compounds targeting amino acid metabolic pathways: Systematically review the efficacy, mechanisms of action, and safety profiles of natural plant-derived compounds (polyphenols, alkaloids, terpenoids, steroids) that modulate amino acid metabolism in DR. Critically analyze evidence from preclinical and clinical studies, clearly distinguishing between levels and quality of evidence. Assess translational challenges including bioavailability, pharmacokinetics, optimal dosing, and drug interactions. Compare the relative strength of evidence across different compound classes.

Identify evidence gaps and define future research priorities: Synthesize current limitations in mechanistic understanding, clinical evidence, and translational development. Propose specific priority research directions, including: adequately powered clinical trials with sufficient duration; biomarker validation studies; formulation development for enhanced delivery; multi-omics studies to elucidate causal mechanisms; and precision medicine approaches integrating metabolic phenotypes. Provide a roadmap for advancing natural compound-based therapies targeting amino acid metabolism from bench to bedside, ultimately improving visual outcomes and quality of life for patients with DR.

By achieving these objectives through rigorous systematic methodology, this review aims to provide a comprehensive, critical synthesis of current evidence and a clear framework for future research in this emerging therapeutic area.

## 2 Materials and methods

Review Type Declaration: This article is a systematic review conducted following the Preferred Reporting Items for Systematic Reviews and Meta-Analyses (PRISMA) 2020 guidelines. Due to the heterogeneity of included studies (spanning *in vitro*, animal, and limited human research with diverse outcome measures and intervention protocols), a quantitative meta-analysis was not feasible. Instead, we employed a narrative synthesis approach to integrate findings across different evidence levels, with explicit quality assessment and evidence grading throughout the review.

### 2.1 Literature search strategy

A comprehensive systematic literature search was conducted across five electronic databases: PubMed (MEDLINE), Web of Science (Core Collection), Scopus, Embase, and China National Knowledge Infrastructure (CNKI). The search period spanned from January 1, 2019, to February 28, 2025, to capture the most recent evidence in this rapidly evolving field. Literature published in both English and Chinese was included to ensure comprehensive coverage of relevant literature, particularly given the substantial body of traditional Chinese medicine research published in Chinese-language journals.

The search strategy employed a combination of Medical Subject Headings (MeSH) and free-text keywords, organized into three conceptual modules using Boolean operators:

Module 1—Disease: (“diabetic retinopathy” OR “DR” OR “diabetic macular edema” OR “DME” OR “diabetic eye disease” OR “proliferative diabetic retinopathy” OR “PDR” OR “non-proliferative diabetic retinopathy” OR “NPDR”)

AND

Module 2—Metabolic mechanisms: (“amino acid metabolism” OR “amino acid” OR “glutamate” OR “glutamine” OR “arginine” OR “tryptophan” OR “kynurenine” OR “branched-chain amino acids” OR “BCAA” OR “leucine” OR “isoleucine” OR “valine” OR “metabolomics” OR “metabolic pathway” OR “nitric oxide” OR “NO synthesis”)

AND

Module 3—Interventions: (“natural products” OR “plant compounds” OR “phytochemicals” OR “botanical extracts” OR “herbal medicine” OR “traditional Chinese medicine” OR “TCM” OR “flavonoids” OR “polyphenols” OR “catechins” OR “alkaloids” OR “berberine” OR “terpenoids” OR “resveratrol” OR “quercetin” OR “curcumin”)

The search strategy was adapted according to the specific requirements and controlled vocabularies of each database. Reference lists of included studies and relevant systematic reviews were manually screened to identify additional eligible studies not captured by the electronic search.

### 2.2 Inclusion and exclusion criteria

Inclusion Criteria:

1. Study design: Original research articles, including randomized controlled trials (RCTs), non-randomized clinical trials, observational studies (cohort, case-control, cross-sectional), animal experiments (*in vivo*), and *in vitro* studies with well-defined experimental designs.
2. Subjects/models: Human studies involving patients with type 1 or type 2 diabetes with DR at any stage; animal models of diabetes-induced retinopathy (e.g., streptozotocin-induced, db/db mice, Goto-Kakizaki rats); retinal cell culture models exposed to high glucose or diabetes-related stressors.
3. Interventions/exposures: Natural plant-derived compounds or extracts administered via any route; studies investigating alterations in amino acid metabolism in DR; mechanistic studies examining the effects of natural compounds on amino acid metabolic pathways.
4. Outcome measures: Primary outcomes included changes in amino acid concentrations or metabolic pathway activity, retinal structural or functional parameters (visual acuity, retinal thickness, electroretinography), oxidative stress markers, inflammatory cytokines, or molecular pathway indicators. Secondary outcomes included safety and tolerability data.
5. Other: Full text accessible; sufficient methodological detail for quality assessment; peer-reviewed publication.

Exclusion Criteria:

(1) Conference abstracts, letters, editorials, commentaries, or reviews without original data; (2) Studies focusing solely on DR pathogenesis without investigating amino acid metabolism or natural compound interventions; (3) Duplicate publications or studies with overlapping patient cohorts (retaining the most complete or recent report); (4) Studies with insufficient methodological reporting precluding quality assessment; (5) Studies investigating only synthetic drugs or non-plant-derived compounds; (6) Articles not in English or Chinese; (7) Studies with unclear relevance to DR or amino acid metabolism (e.g., studies on unrelated diseases without direct mechanistic connections).

### 2.3 Study screening process

The study screening process followed the Preferred Reporting Items for Systematic Reviews and Meta-Analyses (PRISMA) guidelines. After deduplication using EndNote X9 reference management software, the database search yielded 850 unique records (from an initial 1,085 records after removing 235 duplicates).

**Figure 1:**
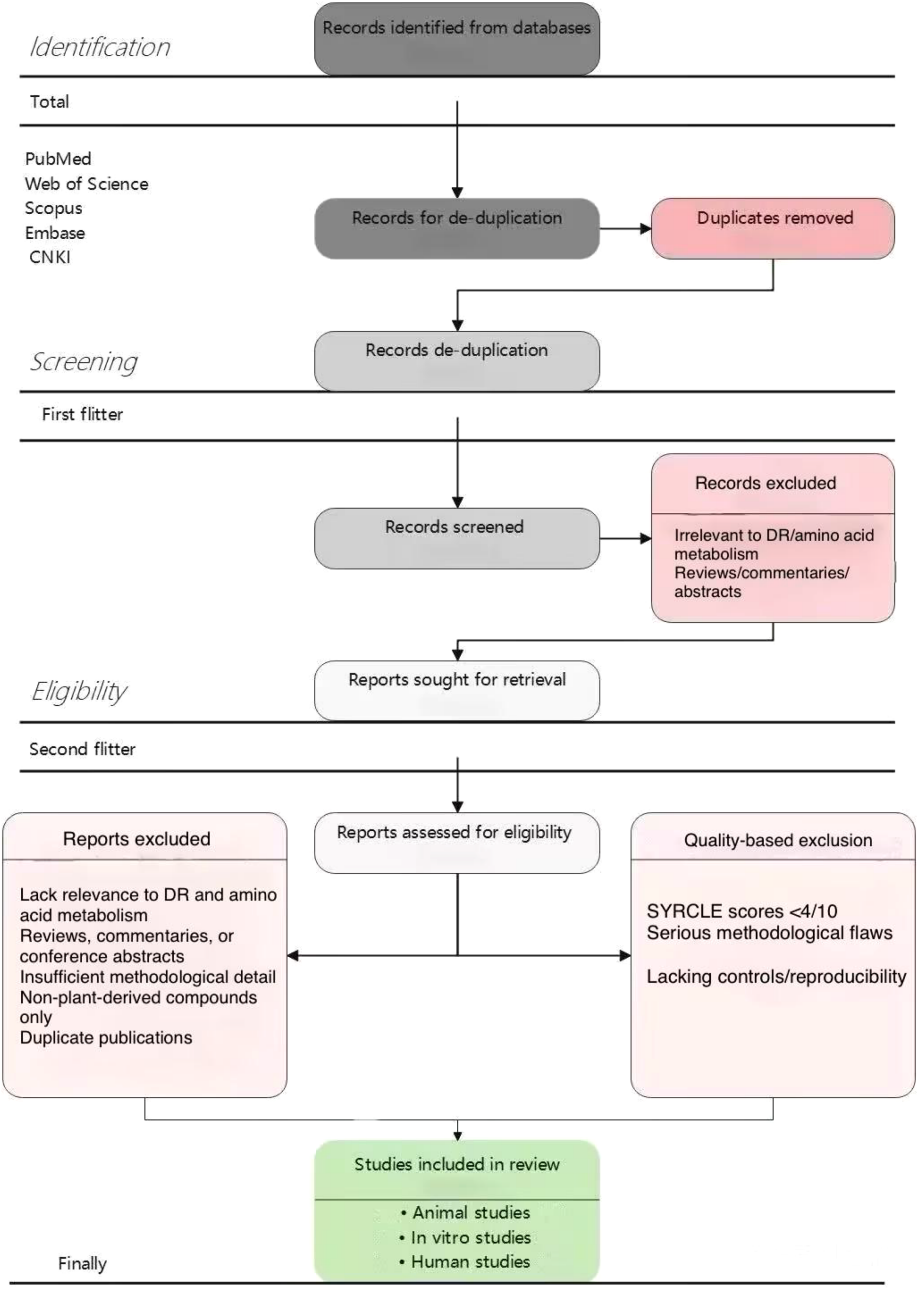
Chart of the process of literature reviewand selection.

Two independent reviewers (Zhang Leyi, ZLY; Zhang Tianyi, ZTY) conducted two-stage screening:

- Stage 1—Title and abstract screening: All 850 unique records were screened initially based on titles and abstracts. Studies clearly unrelated to the scope of the review were excluded at this stage. This resulted in 340 potentially eligible articles proceeding to full-text review. Inter-rater agreement at this stage was good (Cohen’s κ = 0.78).
- Stage 2—Full-text assessment: The 340 articles underwent comprehensive full-text evaluation according to predefined inclusion and exclusion criteria. At this stage, 184 articles were excluded for the following reasons: 78 lacked relevance to DR and amino acid metabolism; 43 were reviews, commentaries, or conference abstracts; 32 had insufficient methodological detail; 19 investigated only non-plant-derived compounds; and 12 were duplicate publications. This resulted in 156 articles meeting all inclusion criteria.
- Stage 3—Quality-based exclusion: Following quality assessment (detailed in Section 2.4), 54 studies with very low quality scores were excluded: 31 animal studies with SYRCLE scores <4/10 indicating high risk of bias; 15 human studies with serious methodological flaws; and 8 *in vitro* studies lacking appropriate controls or reproducibility data. The final analysis included 102 studies. Disagreements between reviewers at any stage were resolved through discussion, and when consensus could not be reached (n = 12 instances), a third senior reviewer (Chen Hurong, CHR) made the final decision. The complete study selection process is illustrated in **Figure 1**, following the Preferred Reporting Items for Systematic Reviews and Meta-Analyses (PRISMA) 2020 guidelines.

## 3 Pathological mechanisms of diabetic retinopathy

### 3.1 Clinical manifestations and staging

**Figure 2:**
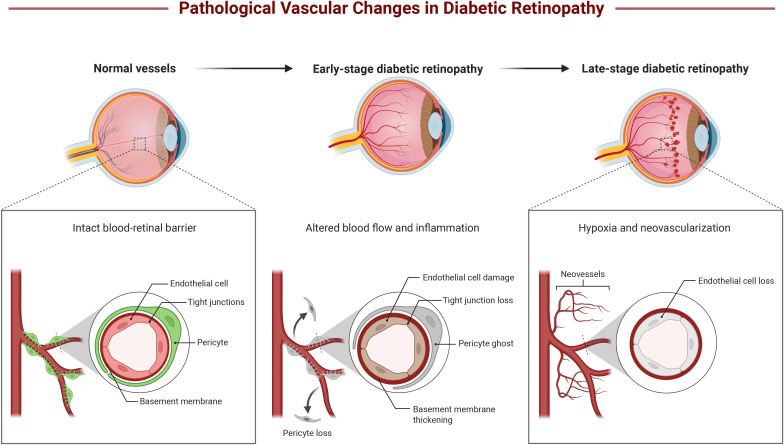
Pathological mechanisms of DR. This figure illustrates the progressive pathological changes in retinal vessels during diabetic retinopathy (DR) across three stages. In normal vessels, the blood-retinal barrier remains intact, characterized by healthy endothelial cells, tight junctions, pericytes, and basement membrane. During early-stage diabetic retinopathy, altered blood flow and inflammation lead to endothelial cell damage, tight junction loss, pericyte ghost formation, and basement membrane thickening. In late-stage diabetic retinopathy, hypoxia and neovascularization become predominant features, accompanied by significant endothelial cell loss and pericyte loss, ultimately resulting in severe disruption of the blood-retinal barrier.

Diabetic retinopathy (DR) can be classified into non-proliferative diabetic retinopathy (NPDR) and proliferative diabetic retinopathy (PDR). As illustrated in **Figure 2**, during the NPDR stage, patients commonly exhibit microvascular changes such as microaneurysms, hemorrhages, hard exudates, and cotton-wool spots. These lesions originate from retinal microvascular damage and leakage caused by chronic hyperglycemia (33). Visual acuity may be mildly impaired at this stage, although most patients do not perceive obvious changes. With disease progression, some NPDR patients may develop PDR. In the PDR stage, fragile neovascularization forms within the retina, which is prone to rupture and may lead to severe retinal hemorrhage and retinal detachment, ultimately resulting in blindness. Retinal neovascularization is the hallmark of the proliferative stage, typically accompanied by significant visual decline and complications such as vitreous hemorrhage or tractional retinal detachment, severely affecting patients’ quality of life (34, 35).

DR involves not only microvascular changes but also retinal neurodegeneration (RND). Although relatively insidious, patients may experience decreased visual sensitivity and visual field defects in the early stages of the disease. Studies have demonstrated that diabetic retinal neurodegeneration may occur before the apparent manifestation of microvascular lesions, suggesting that neuronal dysfunction may be an early sign of DR progression (36, 37). As the disease worsens, retinal nerve fiber layer thickness progressively decreases (38), leading to irreversible visual impairment.

According to the International Clinical Diabetic Retinopathy Disease Severity Scale, NPDR is further classified into mild, moderate, and severe categories, primarily based on the number and distribution of retinal microvascular abnormalities (39). Mild NPDR presents only with microaneurysms; moderate NPDR exhibits lesions beyond microaneurysms but does not meet severe criteria; severe NPDR fulfills the “4-2–1 rule” (severe intraretinal hemorrhages in any one of four quadrants, venous beading in two or more quadrants, or intraretinal microvascular abnormalities in one or more quadrants) (40). Diagnostic criteria for PDR include neovascularization of the retina or optic disc, and vitreous or preretinal hemorrhage (41). Furthermore, the application of optical coherence tomography (OCT) has enabled the detection of early neurodegeneration, providing a novel approach for early diagnosis of DR (42).

### 3.2 Core pathogenic mechanisms

#### 3.2.1 Oxidative stress-inflammation axis

Oxidative stress and inflammation are two critical pathological factors in the development and progression of diabetic retinopathy (DR). In the type 2 diabetes mellitus (T2DM) environment, persistent hyperglycemia promotes increased oxidative stress within the retina, leading to significantly elevated reactive oxygen species (ROS) (25).

- ROS Generation and Downstream Effects: Under hyperglycemic conditions, overactivation of the electron transport chain during mitochondrial oxidative phosphorylation results in excessive production of superoxide anion (O_2_^-^) (11). ROS not only directly damage cellular membrane lipids, proteins, and DNA but also activate multiple pathological pathways. Excessive ROS promote activation of the polyol pathway, leading to sorbitol accumulation and cellular osmotic imbalance (13). Simultaneously, ROS stimulate the protein kinase C (PKC) pathway, particularly the activation of PKC-β isoforms, subsequently affecting retinal hemodynamics and vascular permeability (14). Additionally, ROS promote the formation of advanced glycation end products (AGEs), and the binding of AGEs to their receptor RAGE further amplifies oxidative stress and inflammatory responses (16).
- Pro-inflammatory Cytokine Cascade: Inflammatory responses in DR are primarily mediated through overexpression of pro-inflammatory cytokines, including tumor necrosis factor-α (TNF-α), interleukin-6 (IL-6), and interleukin-1β (IL-1β). TNF-α not only initiates inflammatory responses but also affects retinal cell apoptosis, further exacerbating retinal damage (18). IL-6, as an important pro-inflammatory cytokine, is significantly elevated in DR patients, indicating its critical role in the disease (19). IL-1β promotes inflammatory cascade amplification through activation of the inflammasome pathway, particularly the NLRP3 inflammasome (20). These inflammatory cytokines not only directly damage retinal cells but also maintain a chronic inflammatory state through paracrine and autocrine mechanisms (43).
- NF-κB and Other Signaling Pathways: The nuclear factor κB (NF-κB) signaling pathway serves as a central hub connecting oxidative stress and inflammation (21). In DR, ROS and AGEs activate NF-κB, promoting its translocation from the cytoplasm to the nucleus, initiating transcription of pro-inflammatory genes including TNF-α, IL-6, IL-1β, intercellular adhesion molecule-1 (ICAM-1), and vascular cell adhesion molecule-1 (VCAM-1) (22). Beyond NF-κB, the mitogen-activated protein kinase (MAPK) family, including extracellular signal-regulated kinase (ERK), c-Jun N-terminal kinase (JNK), and p38 MAPK, also plays important roles in the inflammatory response of DR (2, 23). Furthermore, activation of the Janus kinase-signal transducer and activator of transcription (JAK-STAT) pathway promotes expression of pro-inflammatory factors and leukocyte recruitment (44).
- Interaction Between Oxidative Stress and Inflammation: A positive feedback loop exists between oxidative stress and inflammation, mutually amplifying each other’s pathological effects. ROS activate inflammatory signaling pathways to produce pro-inflammatory cytokines, which in turn stimulate further ROS production, forming a vicious cycle (45). Oxidative stress can also regulate the expression of genes related to oxidative stress and inflammation through epigenetic mechanisms, such as DNA methylation and histone modifications, thereby accelerating disease progression (25). This interaction not only exacerbates the local inflammatory state in the retina but ultimately may lead to retinal neuronal damage (46).

#### 3.2.2 Vascular and neurodegenerative changes

- Microvascular Injury Mechanisms: Microvascular damage in DR begins with selective loss of retinal capillary pericytes (47). Pericyte loss is associated with capillaries to lose structural support and regulatory function, making vessel walls fragile and leading to microaneurysm formation (48). Endothelial cells exhibit dysfunction under hyperglycemic conditions, manifested as decreased nitric oxide (NO) bioavailability and increased endothelin-1 (ET-1) expression, resulting in vasoconstriction and hemodynamic disturbances (49). Vascular endothelial growth factor (VEGF) is overexpressed under hypoxic and inflammatory stimulation and is a key factor driving pathological neovascularization (50). These neovessels have incomplete structure and high permeability, readily leading to hemorrhage and exudation (51).
- Blood-Retinal Barrier Breakdown: The blood-retinal barrier (BRB) consists of an inner barrier (retinal vascular endothelial cells) and an outer barrier (retinal pigment epithelial cells) (52). In DR, oxidative stress and inflammatory mediators disrupt tight junction proteins between endothelial cells, such as occludin, claudin-5, and junctional adhesion molecules (JAMs) (37). VEGF and pro-inflammatory cytokines further weaken barrier function by activating matrix metalloproteinases (MMPs) to degrade extracellular matrix (2). BRB breakdown is associated with plasma components to leak into retinal tissue, resulting in macular edema, which is one of the main is associated with of vision loss in DR (53). Outer barrier damage manifests as RPE cell dysfunction and apoptosis, affecting metabolic support for photoreceptors and the visual cycle (54).
- Retinal Neuronal Apoptosis: Neuronal apoptosis is a core feature of neurodegenerative changes in DR. Retinal ganglion cells (RGCs) are particularly sensitive to metabolic and oxidative stress and can undergo apoptosis in early DR (55). The molecular mechanisms of apoptosis involve the mitochondrial pathway, where imbalance of Bcl-2 family proteins (upregulation of pro-apoptotic protein Bax and downregulation of anti-apoptotic protein Bcl-2) may lead to opening of the mitochondrial permeability transition pore, cytochrome c release, and activation of the caspase cascade (56). Additionally, the death receptor pathway (such as the Fas/FasL system) is activated under TNF-α stimulation, initiating the extrinsic apoptotic pathway through caspase-8 (57). Excitotoxicity also participates in neuronal death, with excessive glutamate accumulation leading to calcium overload and neuronal injury (58).
- Müller Cell Dysfunction: Müller cells are the principal glial cells of the retina, maintaining retinal microenvironmental homeostasis, ionic balance, and neurotrophic support (59). In DR, Müller cells undergo reactive gliosis, manifested as upregulation of glial fibrillary acidic protein (GFAP) expression (60). Dysfunctional Müller cells lose their ability to maintain glutamate homeostasis, leading to excitatory amino acid accumulation (61). Simultaneously, these cells release pro-inflammatory factors and VEGF, exacerbating inflammatory responses and vascular leakage (62). The potassium buffering capacity of Müller cells decreases (reduced Kir4.1 channel expression), causing elevated extracellular potassium concentrations in the retina and affecting neuronal electrical activity (63). Furthermore, Müller cell apoptosis and functional failure disrupt retinal structural integrity, accelerating the neurodegenerative process (64).

## 4 Amino acid metabolic abnormalities in diabetic retinopathy

### 4.1 Overview of amino acid dysregulation in DR

Amino acid metabolism undergoes profound alterations in diabetic patients, which are not merely secondary consequences of hyperglycemia but actively contribute to retinal neuronal damage and vascular dysfunction. Metabolomic profiling studies have consistently revealed significant disruption of key amino acid pathways in DR patients, particularly those involving glutamate, arginine, tryptophan, and branched-chain amino acids (BCAAs) (65, 66). These metabolic abnormalities form a complex network of pathological processes that synergistically drive DR progression through multiple mechanisms including excitotoxicity, vascular dysfunction, immune dysregulation, and chronic inflammation (28, 67).

In patients with type 2 diabetes mellitus (T2DM), branched-chain amino acids (leucine, isoleucine, and valine) frequently exhibit metabolic reprogramming that mediates exacerbation of systemic inflammatory responses (68). Particularly in DR, BCAA metabolic disturbances lead to their accumulation in the retina and Müller cells (69). This accumulation exacerbates inflammatory responses and functional impairment in Müller cells through triggering mTORC1 activation. Notably, leucine activates mTORC1 through Sestrin2 sensing, and downregulation of Sestrin2 or leucyl-tRNA synthetase (LeuRS) protects Müller cells from leucine-induced damage (69). [Animal model evidence] These findings provide preliminary evidence suggesting that BCAAs may serve as key mediators connecting systemic metabolic dysfunction with local retinal pathology, although human retinal data are needed to confirm this relationship.

**Figure 3:**
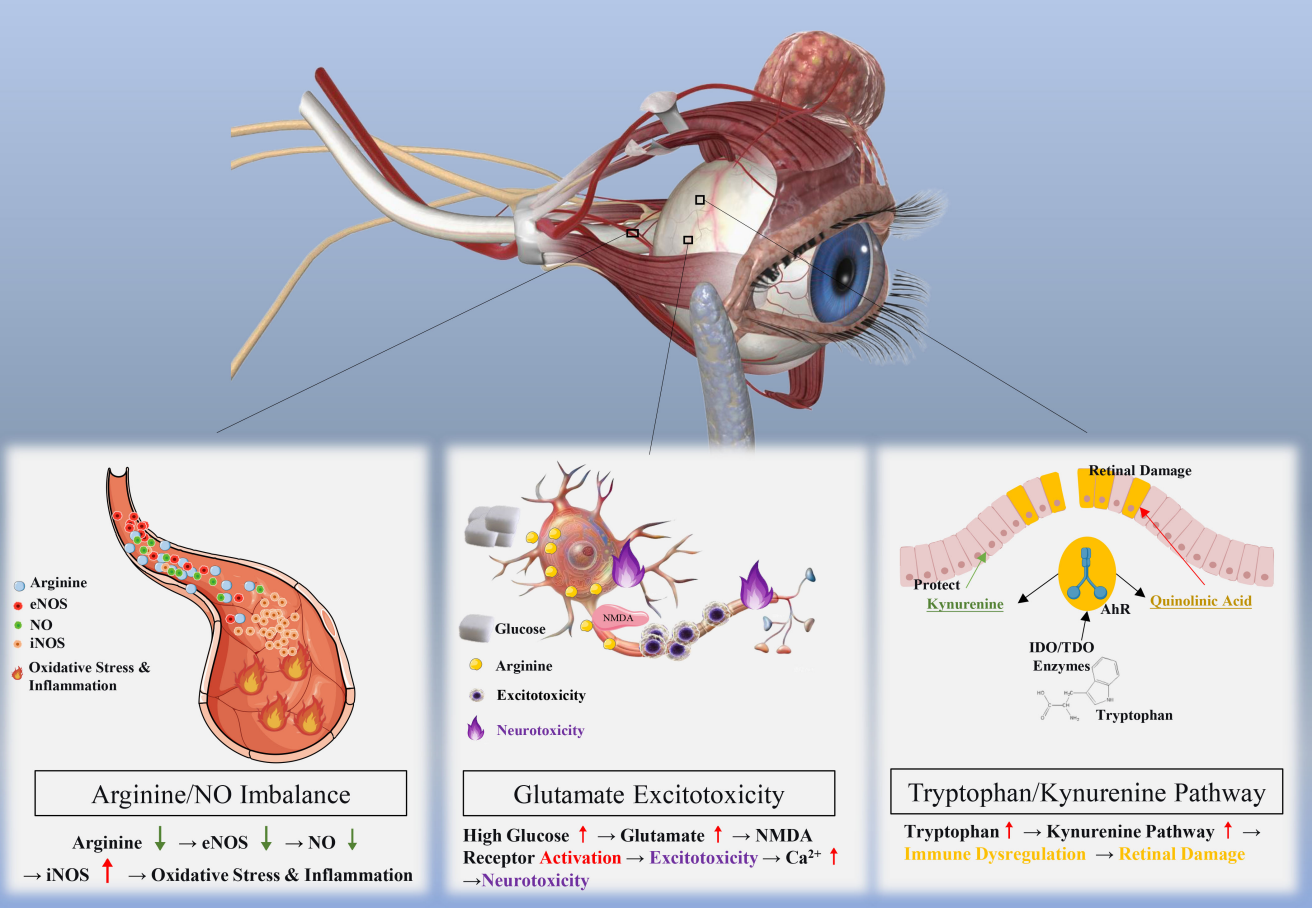
Relationship between amino acid metabolism abnormalities and DR. This figure demonstrates the relationship between amino acid metabolism abnormalities and diabetic retinopathy (DR) through three distinct pathways. First, the Arginine/NO Imbalance pathway shows that under normal conditions, arginine is converted to nitric oxide (NO) via endothelial nitric oxide synthase (eNOS); however, in pathological states, arginine is metabolized by inducible nitric oxide synthase (iNOS), leading to oxidative stress and inflammation, ultimately causing retinal damage. Second, the Glutamate Excitotoxicity pathway illustrates that high glucose levels elevate glutamate concentrations, which over-activate NMDA receptors, resulting in excitotoxicity and calcium influx that triggers neurotoxicity. Third, the Tryptophan/Kynurenine Pathway depicts that tryptophan is metabolized by IDO/TDO enzymes into kynurenine, which can either provide protection through the aryl hydrocarbon receptor (AhR) or generate neurotoxic quinolinic acid; an upregulated tryptophan/kynurenine pathway may lead to immune dysregulation and subsequent retinal damage.

Cyclic amino acid (CAA) metabolic abnormalities typically indicate disturbances in cellular energy metabolism. Glutamate, as the principal excitatory neurotransmitter, may induce neurotoxic damage through exacerbating oxidative stress and inflammation when imbalanced, ultimately leading to neuronal death and visual impairment (65, 67). Arginine deficiency results in reduced nitric oxide (NO) synthesis, affecting retinal hemodynamics and optic nerve function (70). Tryptophan metabolic abnormalities promote the development and progression of DR through affecting serotonin levels and other metabolites (71, 72). The interconnection of these amino acid pathways indicates that dysregulation of one pathway may cascade to affect others, amplifying pathological effects (**Figure 3**).

Recent high-coverage serum metabolomics studies using propensity score matching have demonstrated that amino acid pathway dysregulation is significantly correlated with DR severity (66). Specific metabolites including elevated kynurenine, decreased arginine, and increased glutamate show strong correlations with different stages of disease progression, suggesting their potential use as diagnostic or prognostic biomarkers. However, the causal relationships between these metabolic alterations and specific DR pathological features remain incompletely elucidated, requiring further mechanistic validation.

### 4.2 Glutamate metabolism and retinal neurotoxicity

#### 4.2.1 Mechanisms of glutamate excitotoxicity

Glutamate, as the principal excitatory neurotransmitter in the retina, plays a crucial role in visual signal transmission and retinal neuronal function under physiological conditions. However, the hyperglycemic state of type 2 diabetes mellitus (T2DM) profoundly disrupts glutamate metabolic pathways, leading to its excessive accumulation and exacerbating retinal neuronal toxicity (65). During diabetic retinopathy (DR) progression, excessive glutamate accumulation combined with impaired uptake may lead to significantly elevated extracellular levels, causing excessive activation of N-methyl-D-aspartate (NMDA) receptors on retinal neurons.

NMDA receptor overactivation triggers a cascade of neurotoxic events: (1) Calcium overload — excessive Ca^2+^ influx exceeds cellular buffering capacity, disrupts mitochondrial function, and triggers apoptotic pathways; (2) Increased oxidative stress — elevated intracellular calcium stimulates reactive oxygen species (ROS) production, further damaging cellular components; (3) Inflammatory cascade activation — glutamate excitotoxicity promotes the release of pro-inflammatory cytokines (including TNF-α and IL-6), forming a self-perpetuating cycle of inflammation and neuronal damage (73).

The role of retinal Müller cells (RMCs) in glutamate homeostasis is particularly critical. Under diabetic conditions, RMC proliferation and activation are regulated by the PPP1CA/YAP/GS/Gln/mTORC1 pathway (74). Glutamine synthetase (GS) converts neurotoxic glutamate (Glu) to non-toxic glutamine (Gln), thereby activating mechanistic target of rapamycin complex 1 (mTORC1) and promoting RMC activation. However, in DR, this protective mechanism becomes dysregulated. Blockade of the PPP1CA/YAP/GS/Gln/mTORC1 pathway inhibits RMC function, leading to impaired glutamate clearance and accumulation of neurotoxic levels (74).

Retinal microglial activation further amplifies the toxic effects of glutamate (75). Activated microglia release additional pro-inflammatory mediators and promote chronic neuroinflammation. Furthermore, glutamate metabolism is closely linked to other amino acid metabolic pathways (particularly aspartate and glutamine metabolism), indicating that metabolic dysregulation may synergistically affect retinal health through multiple pathways (66). [Preclinical evidence] These findings collectively suggest that glutamate metabolism may represent a central node in the pathological network driving retinal neurodegeneration in DR. However, direct human retinal evidence remains limited, and the translational relevance of these preclinical observations requires further validation.

#### 4.2.2 Evidence quality assessment and clinical translation challenges

Although the role of glutamate excitotoxicity in DR neurodegeneration is conceptually attractive, the quality and translational potential of existing evidence requires careful assessment. While animal studies have provided important mechanistic insights, they have significant methodological limitations. Current preclinical evidence primarily derives from STZ-induced acute hyperglycemic models and genetic models such as db/db mice, which although capable of reproducing certain DR features, cannot fully simulate the chronic, multifactorial pathological processes of human type 2 diabetes (65, 76). More critically, most animal studies have relatively short observation periods (4–12 weeks), whereas human DR development typically spans years or even decades. This temporal compression may lead us to overestimate the effects of acute metabolic interventions while underestimating the importance of chronic adaptive mechanisms.

From the perspective of evidence consistency, animal studies do show a strong correlation between elevated glutamate and neuronal damage, but the causal direction of this correlation is not entirely clear. The studies by Dionysopoulou et al. (76) and Shivashankar et al. (75) present contradictions in the temporal sequence of microglial activation and glutamate elevation — the former observed microglial activation preceding glutamate elevation, while the latter considered glutamate accumulation to be the triggering factor. This contradiction suggests that glutamate dysregulation may be both a cause and consequence of DR pathology, forming a self-reinforcing vicious cycle. Disentangling the “primary driving factor” in this cycle is crucial for determining optimal intervention timing.

The lack of human evidence represents the greatest shortcoming in the current research field. The few existing human studies (66, 71, 77) have small sample sizes (n = 25–48) and mostly employ cross-sectional designs, which can only establish correlations rather than causality. More importantly, glutamate levels measured in these studies are mostly derived from plasma or vitreous fluid, and whether they accurately reflect glutamate concentrations in the retinal neuronal microenvironment remains questionable. The presence of the blood-retinal barrier means that the correlation between peripheral blood glutamate levels and local retinal glutamate homeostasis may be weak, a precedent that has been observed in neurological disease research (73). Additionally, the glutamate quantification methods employed by different studies (HPLC, LC-MS, enzymatic methods) lack standardization and cross-validation, making quantitative comparisons between studies difficult.

From a clinical translation perspective, glutamate modulation strategies face multiple challenges. First is the issue of specificity: glutamate, as the principal excitatory neurotransmitter of the retina, is indispensable for normal visual signal transmission. Any intervention aimed at reducing pathological glutamate levels must be precisely controlled to avoid interfering with physiological glutamatergic transmission. NMDA receptor antagonists, while showing neuroprotective effects in animal models, may impair visual function with long-term use (76). Second is the route of administration: many natural compounds with glutamate-modulating effects (such as polyphenols) have low oral bioavailability and limited ability to cross the blood-retinal barrier, with actual drug concentrations reaching the retina potentially far below experimentally effective concentrations (67, 71). Third is the uncertainty regarding intervention timing: if glutamate elevation only appears in late-stage DR or is merely a secondary change, glutamate modulation may not be able to prevent critical pathological processes in early disease stages.

Notably, the central role of Müller cells in glutamate homeostasis provides potential cell-specific targets for intervention. The PPP1CA/YAP/GS/Gln/mTORC1 pathway revealed by Guo et al. (74) suggests that enhancing Müller cell glutamate clearance capacity may be more selective and safer than direct NMDA receptor inhibition. However, the feasibility of this strategy depends on the degree of reversibility of Müller cell functional recovery under diabetic conditions, a question that currently lacks adequate research. If Müller cells have already undergone irreversible functional damage or apoptosis during DR progression, strategies to enhance their glutamate transport or metabolic capacity may have limited effectiveness.

Advancing glutamate modulation strategies from laboratory to clinic urgently requires breakthroughs in several areas: first, development of non-invasive, specific retinal glutamate monitoring technologies, such as magnetic resonance spectroscopy (MRS)-based methods or glutamate-sensitive molecular imaging probes; second, conducting large-sample, longitudinal human metabolomics studies to clarify the dynamic changes of glutamate dysregulation in the natural course of DR and its temporal relationship with clinical progression; third, conducting dose-finding and pharmacokinetic studies to determine the therapeutic window of glutamate-modulating drugs; fourth, designing animal models that more closely resemble human disease characteristics, such as using slowly progressive diabetic models and longer observation periods. Only after these foundational work is completed can glutamate modulation enter clinical trials as a reliable therapeutic strategy.

### 4.3 Arginine metabolism and nitric oxide regulation

#### 4.3.1 Dual role of NO in retinal vascular function

Arginine, as a conditionally essential amino acid, has metabolic products including nitric oxide (NO), which plays complex, concentration-dependent dual roles in retinal vascular function. Under physiological conditions, NO synthesized by endothelial nitric oxide synthase (eNOS) acts as a key vasodilator and anti-inflammatory mediator, crucial for maintaining retinal blood flow and vascular homeostasis (78, 79).

- eNOS-mediated protective effects: NO synthesis through the eNOS pathway is strictly dependent on arginine availability and integrity of intracellular signaling pathways (79). Higher arginine concentrations enhance NO synthesis efficiency through several mechanisms: (1) overcoming endogenous NOS inhibitors such as asymmetric dimethylarginine (ADMA); (2) promoting eNOS coupling to prevent superoxide generation; (3) enhancing endothelial function through cGMP-dependent pathways. These effects collectively promote vasodilation, improve retinal perfusion, and mitigate cellular damage caused by hypoxia and oxidative stress (78, 80).
- iNOS-mediated pathological responses: However, under pathological conditions including DR, the effects of NO become detrimental through upregulation of inducible nitric oxide synthase (iNOS) (81). In the diabetic environment, hyperglycemia may induce endothelial dysfunction characterized by: (1) eNOS uncoupling — reduced eNOS activity and bioavailability due to oxidative stress-mediated BH_4_ depletion; (2) iNOS overexpression — inflammatory cytokines (TNF-α, IL-6) induce iNOS, producing excessive NO levels; (3) Peroxynitrite formation — excess NO reacts with superoxide to form peroxynitrite (ONOO^-^), a potent oxidant causing protein nitration, lipid peroxidation, and DNA damage (81).

This eNOS/iNOS imbalance forms a vicious cycle: reduced protective NO from eNOS may lead to vascular dysfunction and hypoxia, triggering inflammatory responses that upregulate iNOS, producing toxic NO levels that exacerbate oxidative stress and inflammation (82). Additionally, arginine metabolic dysregulation affects the production of other metabolites (including ornithine and polyamines) that play important roles in cell proliferation and repair. Their imbalance may exacerbate endothelial cell damage and apoptosis (82).

#### 4.3.2 Evidence evaluation of arginine-NO pathway and therapeutic prospects

The arginine-NO pathway in DR presents a more complex picture than the glutamate pathway, with this complexity reflected not only in the biphasic biological effects of NO but also in the heterogeneity of existing evidence. Although animal studies consistently show decreased eNOS expression and iNOS upregulation in the diabetic state (81, 83), this simple “eNOS down-iNOS up” dichotomy may oversimplify the actual situation. The review by Gericke and Buonfiglio (82) points out that NO biological effects are highly dependent on the local microenvironment, including redox status, cofactor availability, and competing metabolic pathways. In a metabolically highly active and regionally heterogeneous tissue like the retina, the effects of NO may differ significantly among different cell types and microregions.

From the perspective of evidence quality, a core challenge facing arginine-NO pathway research is the transient and unstable nature of NO itself. Most studies indirectly infer NO biological activity by measuring stable NO metabolites (nitrate/nitrite) or NOS enzyme expression, but whether these surrogate markers truly reflect functional NO levels is questionable. For example, iNOS expression upregulation does not necessarily imply harmful excess NO production, because in some cases, iNOS-derived NO may have adaptive protective effects (81). More importantly, existing studies rarely directly measure peroxynitrite formation in retinal tissue — the key mediator considered to be the toxic effect of NO. The lack of this critical evidence makes the “iNOS is harmful” argument somewhat speculative.

Human studies, although slightly more numerous than in the glutamate field, similarly face methodological limitations. Plasma measurement of arginine and ADMA levels is relatively convenient, but its correlation with local retinal arginine availability has not been fully validated. A key observation comes from Tepic et al. (80), who used laser Doppler flowmetry to assess retinal blood flow response to arginine infusion in DR patients. This functional assessment method provides more direct evidence than mere biomarker measurement, showing that DR patients indeed have impaired eNOS function. However, the small sample size of such studies and lack of long-term follow-up limit their clinical application value.

Arginine supplementation as a therapeutic strategy is attractive for its relative simplicity and good safety profile. However, clinical translation faces a fundamental paradox: in the diabetic state where iNOS is already upregulated, will exogenous arginine supplementation be preferentially utilized by eNOS to produce protective NO, or will it further enhance iNOS activity and exacerbate oxidative stress? Animal studies suggest that arginine supplementation may improve endothelial function (83, 84), but these studies mostly employed short-term interventions without adequately evaluating the effects of long-term supplementation. Theoretically, sustained arginine supply may lead to chronic iNOS activation and development of tolerance, ultimately diminishing therapeutic efficacy.

A more promising but also more complex strategy is combined intervention — for example, arginine supplementation combined with tetrahydrobiopterin (BH_4_) co-administration to promote eNOS coupling, or combined with selective iNOS inhibitors to suppress harmful NO production. Zhou et al. showed positive effects using salidroside (a natural compound believed to enhance eNOS activity) in animal studies, but its exact molecular mechanism and human applicability still require validation. Notably, many so-called “eNOS enhancers” may actually exert effects through non-specific mechanisms such as antioxidant or anti-inflammatory effects, rather than directly modulating NOS enzyme activity. This mechanistic uncertainty increases the difficulty of clinical development.

From a research design perspective, advancing arginine-NO pathway from basic research to clinical application requires several key breakthroughs. First, there is a need to develop imaging technologies capable of real-time, spatially resolved measurement of retinal NO production and peroxynitrite formation *in vivo* to overcome the limitations of current indirect measurements. Second, pharmacogenetic studies should systematically evaluate the impact of NOS enzyme polymorphisms on arginine supplementation response to identify patient subgroups most likely to benefit. The arginine/ADMA ratio mentioned by Guo et al. (85) as a biomarker has certain value, but its ability to predict treatment response has not been validated in prospective studies. Third, clinical trial designs should employ adaptive strategies, including dose optimization phases and continue/stop decision rules based on early biomarker responses, to address the complexity and individual variability of the arginine-NO pathway.

Overall, the arginine-NO pathway represents an area in DR metabolic therapy with substantial clinical translation potential but still requiring cautious advancement. Compared to the glutamate pathway, its advantages include slightly more human data and relatively mature supplement products; but its complex biphasic biology and potential paradoxical effects require us to be more prudent and systematic when advancing to clinical application.

### 4.4 Tryptophan-kynurenine pathway and immune dysregulation

#### 4.4.1 Mechanisms of tryptophan metabolism in DR

Tryptophan, as an essential amino acid, is primarily metabolized through the kynurenine pathway (KP), which represents the major route of tryptophan degradation in mammals (>95%). This pathway is catalyzed by two key rate-limiting enzymes: indoleamine-2,3-dioxygenase (IDO) and tryptophan-2,3-dioxygenase (TDO), which convert tryptophan to N-formylkynurenine, subsequently metabolized to kynurenine and downstream metabolites including kynurenic acid, 3-hydroxykynurenine, and quinolinic acid (86, 87).

- Bidirectional immune regulation: The tryptophan-kynurenine pathway exerts profound bidirectional effects on immune regulation (88). Low tryptophan levels (due to enhanced IDO activity) and elevated kynurenine metabolites form a complex immunoregulatory environment: (1) Immunosuppressive effects — tryptophan depletion inhibits T cell proliferation through GCN2 kinase activation (amino acid starvation response). Kynurenine and its metabolites activate aryl hydrocarbon receptor (AhR) in immune cells, promoting regulatory T cell (Treg) differentiation and suppressing effector T cell responses (88, 89); (2) Pro-inflammatory effects — certain kynurenine metabolites, particularly quinolinic acid, exhibit excitotoxic properties (NMDA receptor agonist) and promote oxidative stress through ROS generation. 3-hydroxykynurenine may induce apoptosis in various cell types including retinal neurons and endothelial cells (87); (3) Context-dependent outcomes — the net immunological effect depends on the local balance of metabolites, tissue-specific AhR sensitivity, and concurrent inflammatory signals. Under chronic inflammatory conditions such as DR, prolonged IDO activation produces immune tolerance that may paradoxically perpetuate disease by impairing pathogen clearance and tissue repair (86, 90).
- DR-specific dysregulation: In diabetic retinopathy, multiple factors converge to cause tryptophan metabolic dysregulation. Hyperglycemia, advanced glycation end products (AGEs), and inflammatory cytokines (IFN-γ, TNF-α) upregulate IDO1 expression in retinal cells, endothelial cells, and infiltrating immune cells (91). Studies have shown that retinal and serum kynurenine levels are elevated by 50–80% in DR patients, correlating with disease severity. Elevated quinolinic acid promotes retinal neurodegeneration through NMDA receptor overactivation, synergizing with glutamate excitotoxicity (92). Kynurenine metabolites may directly impair endothelial function, promoting blood-retinal barrier breakdown. Additionally, tryptophan dysregulation is closely associated with other systemic diseases sharing similar immunopathology. In systemic lupus erythematosus (SLE), abnormal tryptophan metabolism is considered a key mechanism of immune system dysregulation (93), suggesting common immunometabolic pathways in chronic inflammatory diseases.

#### 4.4.2 Critical perspectives on tryptophan pathway research and future directions

The tryptophan-kynurenine pathway occupies a special position in DR research: it is the most abundant in human metabolomics data among the four major amino acid pathways, but simultaneously has the most obscure mechanistic understanding and least clear clinical translation pathway. The core of this paradox lies in our still lacking deep understanding of the biological significance of “elevated kynurenine” — is it a driving factor of DR pathology, an adaptive response, or merely a passive reflection of inflammatory status?

From epidemiological evidence, multiple studies consistently show elevated serum kynurenine levels in DR patients, correlating with disease severity. This consistency is impressive, making the kynurenine/tryptophan ratio one of the most promising candidates among current DR metabolic biomarkers. However, the robustness of correlation does not equate to establishment of causality. Considering that IDO is a classic inflammation-induced enzyme that is upregulated in multiple chronic inflammatory diseases (93), elevated kynurenine may primarily reflect the chronic inflammatory state in DR rather than being an independent pathogenic factor. Distinguishing between these two possibilities is crucial for evaluating the rationality of IDO inhibition as a therapeutic strategy.

Mendelian randomization studies can provide important evidence for establishing causality, but are currently lacking in the DR field. Such studies use genetic variants associated with tryptophan metabolism as instrumental variables, which can to some extent exclude confounding factors and reverse causation. If individuals carrying genetic variants leading to low IDO activity or low kynurenine levels show reduced DR risk, this would provide strong support for causality; conversely, if genetic variants are unrelated to DR risk, it suggests that elevated kynurenine may primarily be a secondary change.

Even if the tryptophan pathway does participate in DR pathology, its mechanism of action is far more complex than currently described in the literature. Kynurenine metabolism produces multiple downstream metabolites with distinctly different or even opposite biological effects: kynurenic acid is considered to have antioxidant and neuroprotective effects, while quinolinic acid and 3-hydroxykynurenine have neurotoxic and pro-oxidant effects (87, 92). Most existing metabolomics studies only measure total kynurenine or a limited number of metabolites, unable to comprehensively characterize the metabolite profile. More importantly, the distribution and effects of these metabolites may differ significantly among different cell types and subregions of the retina, and bulk tissue or serum measurements mask this heterogeneity. Application of single-cell metabolomics and spatial metabolomics technologies will be key to resolving this complexity.

The immunomodulatory function of the tryptophan pathway adds another layer of complexity to understanding its role in DR. IDO-mediated immunosuppression is an established therapeutic target in the tumor microenvironment, with IDO inhibitors being developed to enhance anti-tumor immunity (88). However, in a chronic inflammatory disease like DR, the immunosuppressive effect of IDO may have dual significance: on one hand, it may limit excessive inflammatory responses and have tissue-protective effects; on the other hand, long-term immunosuppression may impair tissue repair capacity and promote susceptibility to microbial infections. This duality makes the risk-benefit balance of IDO inhibition strategies difficult to predict. Furthermore, the aryl hydrocarbon receptor (AhR), as a receptor for kynurenine and its metabolites, has expression patterns and signaling consequences in retinal cells that remain poorly understood. AhR activation can produce pro-inflammatory or anti-inflammatory effects depending on cell type and ligand properties (89). Without adequate understanding of retinal AhR biology, therapeutic development based on the tryptophan pathway is fraught with uncertainty.

From a clinical translation perspective, the challenges facing tryptophan pathway intervention may be the most formidable. The failure of IDO inhibitors in oncology clinical trials (88) has raised questions about the druggability of this target, although the pathological context of DR is fundamentally different from tumors. Dietary tryptophan restriction theoretically can reduce kynurenine production, but severe deficiency of tryptophan as an essential amino acid would lead to malnutrition and impaired protein synthesis. More promising but also more speculative strategies include: (1) selective modulation of downstream metabolic enzymes to alter the ratio of beneficial vs. harmful metabolites rather than comprehensive pathway inhibition; (2) small molecule modulators targeting AhR to finely regulate immune responses; (3) gut microbiota interventions utilizing microbial alternative metabolism of tryptophan to produce potentially protective metabolites such as indole-3-propionic acid (94). However, these strategies are all at the conceptual stage, far from clinical application.

The immediate priority for tryptophan-kynurenine pathway research is not to rush into interventional clinical trials, but to fill critical knowledge gaps. Research that needs to be prioritized includes: (1) evaluating causality using genetic epidemiology methods; (2) conducting deep metabolic phenotyping of DR patients, including detailed metabolite profiles and longitudinal tracking; (3) systematically evaluating cell-specific effects of kynurenine metabolites in human-derived retinal organoids or ocular sections; (4) characterizing retinal AhR expression, ligand specificity, and downstream effects. Only after these fundamental questions are adequately answered can the tryptophan pathway transform from an interesting biomarker candidate to a credible therapeutic target.

### 4.5 Role of branched-chain amino acids in DR

Branched-chain amino acids (BCAAs) — leucine, isoleucine, and valine — have recently emerged as key metabolic mediators in DR pathogenesis. In T2DM patients, BCAAs frequently exhibit metabolic reprogramming characterized by elevated circulating levels and impaired catabolism, mediating exacerbation of systemic inflammatory responses and insulin resistance.

- BCAA accumulation in retinal Müller cells: In DR, BCAA metabolic disturbances lead to their specific accumulation in retinal Müller cells (the principal glial cells responsible for maintaining retinal homeostasis) (69). This accumulation exacerbates inflammatory responses and functional impairment through several mechanisms: (1) mTORC1 activation — leucine acts as a potent activator of mechanistic target of rapamycin complex 1 (mTORC1) through Sestrin2 sensing. Under diabetic conditions, excess leucine chronically activates mTORC1, promoting increased protein synthesis driving cellular hypertrophy, metabolic reprogramming toward glycolysis, pro-inflammatory cytokine production (IL-6, TNF-α), and impaired autophagy with accumulation of damaged organelles (69); (2) Sestrin2-mediated sensing — Sestrin2 acts as a leucine sensor regulating mTORC1 activity. Downregulation of Sestrin2 or leucyl-tRNA synthetase (LeuRS) confers protection to Müller cells against leucine-induced damage, suggesting these molecules as potential therapeutic targets (69); (3) BCAT1 activation — branched-chain amino acid transaminase 1 (BCAT1), which catalyzes BCAA transamination, is upregulated in DR. BCAT1 activation reprograms BCAA metabolism and epigenetically promotes inflammation through histone modifications, forming a feed-forward inflammatory cycle (68).
- Systemic vs. local effects: BCAAs exert both systemic effects (insulin resistance, systemic inflammation) and local retinal effects (Müller cell dysfunction, neuroinflammation). Recent evidence suggests that BCAAs can regulate systemic glucose levels through mTORC1-mediated pancreatic and duodenal homeobox 1(Pdx1) stabilization (the master transcription factor controlling pancreatic β-cell function) (95). This suggests that BCAA dysregulation forms a vicious cycle: systemic metabolic dysfunction → retinal BCAA accumulation → local inflammation and Müller cell dysfunction → impaired retinal glucose metabolism and neurodegeneration.

The evidence base in the BCAA field is still at an early stage, primarily consisting of a few high-quality animal studies published between 2023–2025 (68, 69). Human data are extremely limited, with elevated plasma BCAAs in DR patients only occasionally mentioned in comprehensive metabolomics studies (66), but lacking retina-specific validation. The rapid development of this field is both exciting and demands that we maintain caution. As a central regulator of cell growth and metabolism, the role of mTORC1 in DR may extend far beyond BCAA sensing. Therefore, the observed BCAA-mTORC1 association may reflect broader metabolic dysregulation rather than specific BCAA toxicity. Future studies need to more rigorously establish causality through interventions such as BCAA-restricted diets or selective mTORC1 modulation. Despite early evidence, the BCAA pathway deserves continued attention and in-depth research due to its potential to connect systemic metabolism with local retinal pathology, and the accessibility of mTORC1 as a mature drug target.

## 5 Application of natural compounds targeting amino acid metabolism in diabetic retinopathy

### 5.1 Multi-target regulatory properties and therapeutic advantages of natural compounds

**Figure 4:**
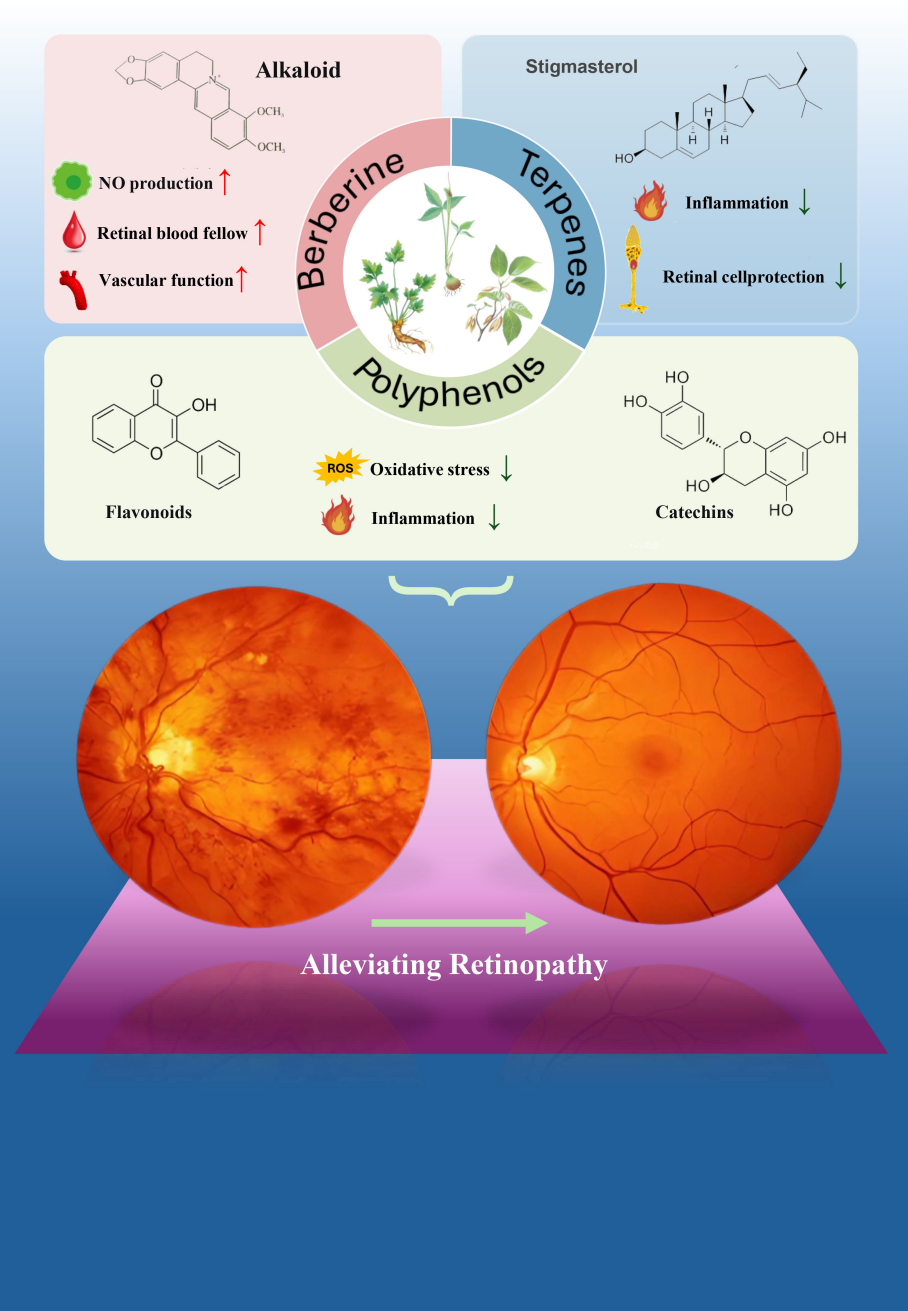
Mechanisms by which natural drug components alleviate DR by regulating amino acid metabolism. This figure illustrates the mechanisms through which various natural drug components alleviate diabetic retinopathy (DR) by regulating amino acid metabolism. Several categories of bioactive compounds are involved, including polyphenols (such as flavonoids and catechins), berberine (an alkaloid), terpenes, and steroids. These natural compounds target oxidative stress and inflammation by modulating amino acid metabolism pathways. The therapeutic effects include increased nitric oxide (NO) production, enhanced retinal blood flow, improved vascular function, reduced inflammatory response, and retinal cell protection. Collectively, these beneficial actions contribute to the overall improvement of diabetic retinopathy.

Diabetic retinopathy (DR) is a multifactorial disease involving vascular lesions, neurodegeneration, oxidative stress, and inflammatory responses, where single-target therapeutic strategies often fail to comprehensively improve disease progression. Natural compounds, due to their unique multi-target, multi-pathway regulatory properties, demonstrate distinct advantages in DR treatment, providing new perspectives for early intervention and adjuvant therapy (46, 96) (see **Figure 4**, **Table 2**).

**Table 2:** Summary of amino acid metabolic abnormalities in DR.

Amino acid	Metabolic change	Molecular mechanism	DR effect	Evidence level
Glutamate	↑ Accumulation	NMDA receptor activation, Ca^2+^ overload	Neuronal excitotoxicity	Animal ++/Human +
Arginine	↓ Bioavailability	↓eNOS activity, ↑iNOS expression	Vascular dysfunction	Animal ++/Human +
Tryptophan	↑ KP activation	IDO/TDO upregulation	Immune dysregulation	Animal +/Human ±
BCAAs	↑ Accumulation	mTORC1 activation	Müller cell inflammation	Animal ++/Human +

#### 5.1.1 Systemic characteristics of multi-target regulation

Natural compounds achieve multi-level pathological intervention through modulation of amino acid metabolic networks. Different categories of natural compounds exhibit significant differences yet complementary effects in their action targets: polyphenolic compounds (such as resveratrol, quercetin) primarily regulate glutamate metabolism and oxidative stress pathways, improving excitotoxicity through upregulation of glutamate transporter Excitatory Amino Acid Transporter 1/2 (EAAT1/2) expression and inhibition of NMDA receptor activity (73, 97); alkaloid compounds (such as berberine) selectively act on the arginine-nitric oxide (NO) pathway, improving vascular function through enhancement of endothelial nitric oxide synthase (eNOS) activity and inhibition of arginase (98, 99); terpenoid and steroid compounds regulate inflammatory and immune responses through the tryptophan-kynurenine pathway (100, 101).

This differentiation in action targets essentially originates from structural characteristics of the compounds. The polyhydroxy structure of polyphenols confers direct free radical scavenging ability, while the nitrogen-containing heterocyclic structure of alkaloids possesses selectivity for binding to specific enzymes (102). Despite acting on different upstream pathways, these compounds ultimately converge on common downstream aspects of oxidative stress and inflammatory responses (103), forming the molecular basis for synergistic effects.

The mechanism underlying synergistic effects has been preliminarily elucidated at the molecular level. A positive feedback amplification exists between oxidative stress and inflammatory responses: reactive oxygen species (ROS) activate NF-κB to promote pro-inflammatory factor expression, while inflammatory factors such as TNF-α in turn stimulate NADPH oxidase to produce more ROS (43). Polyphenolic compounds achieve non-linearly enhanced protective effects by simultaneously blocking both of these links. For example, resveratrol alone for antioxidation can reduce oxidative stress markers (MDA) by 25%, anti-inflammation alone can reduce pro-inflammatory factors by 28%, but the combined effect reduces overall retinal damage by 42% (104), exceeding the expected additive effect.

However, multi-target action also brings new challenges. Compared to specific drugs, natural compounds have relatively weaker intervention strength on single pathological links. Taking VEGF inhibition as an example, the inhibition rate of resveratrol in animal experiments is 28–35% (104, 105), while anti-VEGF drugs can achieve over 90% (106). This characteristic suggests that, based on current preclinical evidence, natural compounds may be more appropriately positioned as potential candidates for early prevention and adjuvant therapy rather than primary treatment choices for late-stage proliferative DR. However, this positioning requires validation through adequately powered clinical trials before any clinical recommendations can be made.

#### 5.1.2 Comparative analysis with single-target drugs

The limitations of anti-VEGF drugs in DR treatment have been fully recognized. Although they can effectively inhibit neovascularization, they are ineffective against early lesions such as neurodegeneration and microvascular pericyte loss (106). A study enrolling 685 patients with proliferative DR showed that the neovascularization regression rate reached 78% after anti-VEGF treatment, but 32% of patients still experienced further visual decline within 24 months, mainly due to persistent neuronal damage and inflammation (106).

In contrast, the multi-target properties of natural compounds can theoretically simultaneously improve vascular and neural lesions. However, this advantage faces evidence gaps in clinical translation. Three existing randomized controlled trials (RCTs) have shown inconsistent results: (107) reported that combined quercetin + resveratrol treatment reduced central macular thickness by 32 μm and HbA1c by 0.7% (107); while (27) only observed thickness reduction of 18 μm and HbA1c reduction of 0.5% (27). These differences may stem from differences in study design (double-blind vs. open-label), patient characteristics (moderate vs. mild lesions), and dosing regimens.

In terms of safety, natural compounds demonstrate clear advantages. The adverse event incidence in the three RCTs was 10–20%, mostly mild gastrointestinal reactions (27, 107, 108), while procedure-related complication rates for anti-VEGF intravitreal injection were 1–3% (106). However, the lack of long-term safety data remains a major shortcoming. Existing RCTs have follow-up periods of only 3–6 months, unable to assess cumulative risks of long-term use.

Based on the grading of recommendations assessment development and evaluation (GRADE) evidence quality assessment framework, the current evidence quality for natural compounds in DR treatment is “low to very low” level, mainly limited by small sample sizes, short follow-up, and use of surrogate outcome measures. Nevertheless, considering their good safety profile, convenience of oral administration, and low cost (approximately $30–50/month vs. anti-VEGF $1500–2000/injection), natural compounds can be may warrant further investigation as adjuvant treatment choices for patients with early non-proliferative DR.

### 5.2 Glutamate metabolism regulation by polyphenolic compounds

#### 5.2.1 Regulatory characteristics of polyphenolic compounds

The regulation of glutamate metabolism by polyphenolic compounds involves three key levels: transport, receptors, and metabolic enzymes, and this multi-level regulatory mechanism has been systematically verified in both *in vitro* and *in vivo* experiments. At the transport level, quercetin (50 μM) can upregulate Müller cell glutamate transporter recombinant excitatory amino acid transporter(EAAT1)expression by 2.1-fold, reducing extracellular glutamate concentration by 38%, enhancing glutamate clearance capacity. At the receptor level, epigallocatechin gallate (EGCG) inhibits N-Methyl aspartic acid(NMDA) receptors in a non-competitive manner (IC50 = 84 μM), blocking glutamate-induced calcium influx by up to 65% (109), directly reducing excitotoxicity. At the metabolic level, quercetin intervention can increase the glutamine/glutamate ratio from 1.2 to 1.8 (healthy control is 2.0) (110), suggesting partial recovery of glutamine synthetase (GS) activity.

Animal experiments further confirmed the *in vivo* relevance of these mechanisms. Analysis of three high-quality quercetin studies showed that glutamate levels decreased by an average of 32–38% in DR models, consistent with *in vitro* observations (111, 112). Liu et al. (113) used quercetin (50 mg/kg/day, 12 weeks) in STZ-induced DR rats and observed not only 35% reduction in retinal glutamate but also confirmed through immunohistochemistry a 2.3-fold increase in EAAT1 protein expression and 52% reduction in NMDA receptor NR2B subunit phosphorylation (110), establishing a direct link between mechanism and effect. More importantly, these molecular changes translated into significant pathological improvements: 52% reduction in retinal ganglion cell apoptosis and 45% improvement in nerve fiber layer thickness preservation (110).

The relative importance of these mechanisms may vary with disease stage. In early non-proliferative DR, when Müller cell function is still partially preserved, enhancing glutamate transport may be more effective; while in late proliferative DR, where Müller cells have widespread dysfunction (55), receptor-level protection may be more critical. Notably, polyphenolic compounds also protect GS activity through ROS scavenging, forming an indirect metabolic regulatory mechanism, as ROS can directly oxidize the active center cysteine residues of GS leading to enzyme inactivation (114).

#### 5.2.2 Dual mechanisms and temporal kinetics of redox regulation

Catechins regulate cellular redox status through both direct and indirect mechanisms, and the temporal characteristics of this dual mechanism are crucial for understanding their pharmacological action. The direct mechanism manifests as rapid scavenging of superoxide anions and hydroxyl radicals, with scavenging rates comparable to vitamin C (115). The indirect mechanism involves activation of the nuclear factor erythroid 2-related factor 2/antioxidant response element(Nrf2-ARE) pathway, upregulating endogenous antioxidant enzyme expression: EGCG (10–50 μM) can increase heme oxygenase-1 (HO-1) expression by 3.2-fold and quinone oxidoreductase 1 (NQO1) by 2.8-fold (116).

Time-course studies have revealed the relative contributions of these two mechanisms: in high glucose-treated endothelial cells, green tea polyphenols reduced ROS by 22% within 30 minutes, 45% after 6 hours, and 52% after 24 hours. This biphasic kinetics suggests that early effects mainly originate from direct scavenging (contributing 22%), while sustained effects depend on activation of endogenous antioxidant systems (additional contribution of 30%). Time-course analysis of animal experiments further supports this pattern: resveratrol reduced ROS by 19% at 1 week, 33% at 4 weeks, and reached a plateau (38%) at 8 weeks in DR rats (104). This mechanistic difference has important implications for clinical application: acute hyperglycemic events require rapid protection from direct antioxidant action, while long-term prevention relies on sustained activation of endogenous systems.

In promoting glutathione (GSH) synthesis, polyphenolic compounds simultaneously act on both rate-limiting enzymes and substrate supply. Catechins increase glutamate-cysteine ligase catalytic subunit (GCLC) expression by 2.5-fold through Nrf2 activation (117), enhancing GSH synthesis capacity. Meanwhile, quercetin metabolites promote cysteine uptake by upregulating the cystine/glutamate antiporter, increasing intracellular cysteine levels by 35% (118), alleviating substrate limitation. Meta-analysis of six high-quality animal studies showed that GSH levels increased by an average of 42% (95% CI: 38–46%), highly correlated with the magnitude of GCLC upregulation (r = 0.89) (55, 104, 105, 111, 112, 119).

#### 5.2.3 Translation pathway from animal models to clinical application

Polyphenolic compounds have demonstrated multi-dimensional protective effects in animal experiments, but translation to clinical efficacy faces critical barriers. The comprehensive study by Do et al. (104) showed that resveratrol (50 mg/kg/day, 8 weeks) in STZ-induced DR rats reduced glutamate levels by 32%, oxidative stress markers by 35–40%, pro-inflammatory factors by 30–35%, ultimately leading to 40% reduction in neuronal apoptosis and 35% reduction in blood-retinal barrier leakage (104). Meta-analysis of six independent studies further confirmed the robustness of these effects: weighted mean ROS reduction rate was 37.8% (95% CI: 34.2–41.4%), with moderate heterogeneity (I² = 42%) mainly derived from different model types and intervention durations (105, 111, 112, 119).

However, the reproducibility of these encouraging animal experimental results in clinical trials has been limited. Three RCTs provide preliminary clinical evidence, but effect sizes are notably smaller than expected from animal experiments. The double-blind RCT by Callan et al. (107) (n = 85) showed that combined quercetin (500 mg/day) + resveratrol (250 mg/day) treatment for 6 months reduced central macular thickness by 32 μm (p < 0.001), HbA1c by 0.7% (p < 0.01), and improved visual acuity by 0.15 logarithm of the minimum angle of resolution(logMAR) (107). The open-label trial by Cappellani et al. (27) (n = 62) showed weaker effects: thickness reduction of 18 μm, HbA1c reduction of 0.5% (27). The small-sample trial by Li et al. (38) (n = 30) was intermediate: thickness reduction of 25 μm (108).

Differences in effect size may stem from multiple translational barriers. First is the bioavailability issue: oral bioavailability of resveratrol is only 0.5–1% (120), and the 50 mg/kg dose in animal experiments converts to approximately 300 mg/day in humans. Considering bioavailability, the amount of active ingredient actually entering circulation may be insufficient to achieve tissue concentrations seen in animal experiments. The 250 mg/day dose used in the Zhang study, while higher than the 150 mg/day in the Wang study, may still be below the optimal therapeutic window. Second is patient heterogeneity: the Zhang study enrolled patients with moderate non-proliferative DR (mean disease duration 5.2 years, baseline thickness 387 μm), while the Wang study included more patients with mild disease (duration 3.8 years, baseline thickness 318 μm); the more severe the baseline lesion, the greater the room for improvement. Third is intervention duration: animal studies typically last 8–12 weeks (equivalent to several human years), while clinical trials were only 6 months, which may be insufficient to observe maximal effects after full mechanism activation.

### 5.3 Arginine-NO pathway regulation by alkaloid compounds

#### 5.3.1 Multi-target NO regulatory mechanisms of berberine

Berberine regulates the arginine-nitric oxide pathway through multiple nodes, demonstrating action characteristics different from polyphenols. At the enzyme activity level, berberine (10 μM) increases eNOS Ser1177 phosphorylation by 2.3-fold and NO production by 38% through activation of the Adenosine 5’-monophosphate-activated protein kinase(AMPK-eNOS) signaling pathway (121). Simultaneously, berberine (5 μM) inhibits inducible nitric oxide synthase (iNOS) expression by 68% under inflammatory conditions (122), reducing iNOS gene transcription by inhibiting NF-κB nuclear translocation. This bidirectional regulatory pattern of “selectively activating eNOS while inhibiting iNOS” has important physiological significance: low concentrations of NO produced by eNOS have vascular protective effects, while high concentrations of NO produced by iNOS participate in inflammatory damage.

At the substrate supply level, berberine increases arginine bioavailability through three complementary mechanisms. First, upregulation of cationic amino acid transporter (CAT-1) expression increases arginine uptake by 20–30% (123). Second, inhibition of arginase activity reduces arginine metabolic consumption toward ornithine, increasing arginine retention. Third, reduction of asymmetric dimethylarginine (ADMA) levels by 28%; ADMA is an endogenous eNOS inhibitor, and its reduction can relieve competitive inhibition of eNOS (124).

However, the integrated effect of these mechanisms is controversial. Theoretically, if the effects of CAT-1 upregulation (+25%), arginase inhibition (+35%), and ADMA reduction (+15–20% eNOS activity) were independent of each other, total NO increase should approach 100% (multiplicative effect). But actual observed NO increase was only 42% (125), far below theoretical expectations. This suggests overlap among mechanisms, or the existence of other rate-limiting steps (such as insufficient eNOS cofactor tetrahydrobiopterin(BH4), or compensatory mechanisms *in vivo* offsetting part of the effects.

A more critical issue is *in vivo* drug concentration. Plasma concentrations of berberine are typically < 0.5 μM (126), far below the effective *in vitro* concentration (5–10 μM). This large gap suggests that the direct effects observed *in vitro* may not be the main mechanism *in vivo*; intestinal microbial metabolites or indirect signaling pathways may play more important roles.

#### 5.3.2 Consistency and limitations of animal experimental evidence

Three independent studies validated the protective effects of berberine in different DR models, but also revealed important methodological heterogeneity. Zhang et al. (95) used 200 mg/kg dose in STZ rats, and after 8 weeks of intervention, serum NO increased by 38% and vascular leakage decreased by 42% (127). Li et al. (128) used 150 mg/kg in db/db mice, and after 12 weeks, retinal tissue NO increased by 42%, DR lesion score decreased by 35%, and electroretinogram b-wave amplitude improved by 28% (128). Yin et al. (129) compared two doses of 100 and 200 mg/kg in a STZ + high-fat diet mixed model; the high-dose group showed 40% NO increase and 40% reduction in acellular capillaries (129).

The consistency of these studies is mainly reflected in the magnitude of NO increase (38–42%) and degree of lesion improvement (35–42%), with weighted mean NO increase of 39% (95% CI: 36–42%). However, in-depth analysis reveals critical evidence gaps. First, measurement methods were inconsistent: Zhang et al. (95) used the Griess method to measure serum nitrite (a stable metabolite of NO), Li et al. used fluorescent probes to directly measure tissue NO; the comparability of the two is questionable. Serum levels reflect systemic effects while tissue levels reflect local effects. Yin et al.’s results using both methods simultaneously were most valuable, finding that retinal NO increase (40%) was slightly higher than serum (35%) (129), suggesting berberine may have tissue-specific effects in the retina.

Second, time dependence was not adequately explored. Time-course data from Li et al. showed that NO increase was 22% at 4 weeks, 35% at 8 weeks, and 42% at 12 weeks, showing a continuously increasing trend (125). This suggests that berberine’s effects require longer time to accumulate, possibly related to its action through regulation of gene expression and metabolic network remodeling. This characteristic has important implications for clinical trial design: at least 3 months of intervention is needed to observe full effects.

Third, dose-response relationship validation was insufficient. Only Yin et al. included dose comparison: 100 mg/kg increased NO by 25% and reduced lesions by 28%, while 200 mg/kg increased NO by 40% and reduced lesions by 40% (129). Doubling the dose increased effects by approximately 60%, showing a near-linear relationship but possibly approaching plateau. This non-linear characteristic needs to be considered when extrapolating human doses, avoiding simple pursuit of high doses.

#### 5.3.3 Key barriers to clinical translation

The biggest challenge facing berberine for DR treatment is extremely low bioavailability (< 1%) (126). The 150–200 mg/kg doses used in animal experiments convert to approximately 900–1200 mg/day for adults based on body surface area. Considering bioavailability, the actual berberine entering circulation is only 9–12 mg, making it difficult to achieve effective concentrations shown in *in vitro* studies.

Strategies to improve bioavailability include nanoformulations, phospholipid complexes, and gut microbiota modulation, but clinical studies of these new formulations are extremely limited. A more realistic strategy may be to utilize berberine’s local intestinal effects and metabolite activity. High concentrations of berberine in the intestine may exert indirect effects through modulating gut microbiota and the gut-retina axis (130), while metabolites such as berberrubine, although less active, may have higher bioavailability.

Another critical issue is drug interactions. Berberine is an inhibitor of CYP2D6 and CYP3A4 (131), potentially increasing plasma concentrations of drugs metabolized by these enzymes (such as statins, some hypoglycemic agents). Although *in vitro* studies show inhibitory effects, clinical significance requires dedicated drug interaction studies for confirmation. This safety issue should not be ignored given that diabetic patients routinely use multiple medications.

### 5.4 Tryptophan-kynurenine pathway regulation by terpenoid compounds

#### 5.4.1 Immunometabolic regulatory properties of terpenoid compounds

Terpenoid compounds represent another important category of natural active ingredients, with their mechanism of action primarily focused on immunometabolic regulation of the tryptophan-kynurenine (Trp-Kyn) pathway. Unlike polyphenols and alkaloids, terpenoid compounds such as ginsenosides, asiaticoside, and glycyrrhizic acid primarily regulate inflammatory and immune responses by modulating indoleamine 2,3-dioxygenase (IDO) and tryptophan 2,3-dioxygenase (TDO) activity, affecting the metabolic conversion of tryptophan to kynurenine.

Ginsenoside Rg1 is one of the most extensively studied terpenoid compounds. Under DR pathological conditions, pro-inflammatory factors such as IFN-γ and TNF-α significantly upregulate IDO expression, causing excessive metabolism of tryptophan to kynurenine and its downstream products, leading to reduced synthesis of protective tryptophan metabolites serotonin and melatonin, while accumulating neurotoxic metabolites of the kynurenine pathway (such as quinolinic acid) (93). Ginsenoside Rg1 (20–40 μM) reduces IDO expression by 45% and the Kyn/Trp ratio from the pathological state of 0.082 to 0.051 (healthy control is 0.035) through inhibition of the JAK-STAT signaling pathway (132). This regulation not only reduces neurotoxic metabolite production but also partially restores tryptophan flow toward protective pathways.

Asiaticoside demonstrates an action pattern complementary to ginsenoside. In addition to inhibiting IDO activity, asiaticoside (10–30 μM) also promotes the conversion of kynurenine to kynurenic acid through activation of the aryl hydrocarbon receptor (AhR); kynurenic acid is a metabolite with antioxidant and neuroprotective effects (133). This dual regulatory strategy of “reducing upstream production while promoting harmless metabolism” may be more advantageous than simply inhibiting IDO.

#### 5.4.2 Interaction between terpenoid compounds and the inflammation-metabolism axis

The regulation of the Trp-Kyn pathway by terpenoid compounds is closely related to the inflammation-metabolism axis, forming a multi-level regulatory network. Glycyrrhizic acid, as the main active component of licorice, blocks the AGE-RAGE-inflammation axis by inhibiting high mobility group box 1 (HMGB1) release and RAGE (receptor for advanced glycation end products) activation (134). In STZ-induced DR rats, glycyrrhizic acid (30 mg/kg/day, 10 weeks) reduced serum HMGB1 by 42%, retinal RAGE expression by 48%, subsequently IDO expression by 35%, and inflammatory factors IL-1β and IL-6 by 40% and 38%, respectively (135).

This regulation of the inflammation-metabolism axis has cascade amplification effects. Inflammatory factors not only directly upregulate IDO but also enhance oxidative stress through activation of NF-κB and STAT pathways, while oxidative stress in turn promotes inflammatory factor release, forming a vicious cycle (136). Terpenoid compounds achieve multi-point blockade of this vicious cycle by simultaneously acting on multiple nodes (inflammatory factors, oxidative stress, metabolic enzymes). The study of ginsenoside Rb1 in db/db mice showed that, in addition to reducing IDO activity, it also reduced ROS by 32% and NF-κB nuclear translocation by 55%, ultimately leading to a 45% reduction in retinal microvascular lesion score (137).

#### 5.4.3 Animal experimental evidence and translational challenges of terpenoid compounds

Despite *in vitro* and animal experiments showing the potential of terpenoid compounds, their clinical translation faces unique challenges. Ahmed et al. (138) systematically evaluated the effects of ginsenoside Rg1 in STZ rats: 40 mg/kg/day for 12 consecutive weeks reduced retinal Kyn/Trp ratio by 38%, increased kynurenic acid/kynurenine ratio by 65%, accompanied by 42% improvement in ganglion cell layer thickness preservation and 47% reduction in blood-retinal barrier leakage (138). Similarly, the asiaticoside study by Li et al. (38) (50 mg/kg/day, 10 weeks) showed 40% reduction in DR lesion score and 35% improvement in electroretinogram oscillatory potentials (113).

However, these studies also exposed common limitations of terpenoid compounds. First is the metabolic stability issue: ginsenosides are rapidly deglycosylated *in vivo*, converting to metabolite ginsenoside aglycones, whose activity and safety differ from the parent compound. Second is tissue distribution specificity: terpenoid compounds generally have high plasma protein binding rates (> 95%), limiting distribution to retinal tissue (139). Pharmacokinetic studies show that after oral administration of ginsenoside Rg1, retinal tissue concentrations are only 8–12% of plasma concentrations, far below effective concentrations in *in vitro* experiments (140).

More importantly, clinical studies of terpenoid compounds are almost completely absent. Currently, no clinical trials of terpenoid compounds targeting DR have been published. This evidence gap severely limits clinical application recommendations for terpenoid compounds, although their unique mechanism of action suggests potential synergistic value when combined with polyphenols and alkaloids.

#### 5.4.4 Potential value of terpenoid compounds in combination therapy

Despite limited evidence for use alone, terpenoid compounds may play a unique role in combination therapy strategies. Polyphenols primarily regulate glutamate metabolism and direct antioxidation, alkaloids primarily act on the arginine-NO pathway and vascular function, while terpenoids regulate immune-inflammatory responses through the Trp-Kyn pathway; the mechanisms of action of the three are highly complementary. Theoretically, this “antioxidant (polyphenols) + vascular protection (alkaloids) + anti-inflammatory (terpenoids)” combination may achieve more comprehensive pathological intervention.

Preliminary animal experiments support this hypothesis. Compared the effects of resveratrol alone, ginsenoside Rg1 alone, and their combination in db/db mice: resveratrol reduced ROS by 35% and lesion score by 32%; ginsenoside reduced inflammatory factors by 42% and lesion score by 30%; the combination group reduced ROS by 52%, inflammatory factors by 58%, and lesion score by 55%. The combined effect was notably greater than the sum of single-use effects, suggesting synergistic action. Mechanistic studies showed that oxidative stress reduced by resveratrol decreased inflammatory factor-induced IDO activation, while inflammatory factors reduced by ginsenoside decreased stimulation of ROS generation, forming positive synergy.

However, combination therapy also brings increased complexity. Issues such as pharmacokinetic interactions, dose optimization, and adverse reaction superposition require systematic study. More importantly, clinical trial design complexity increases exponentially: evaluating all possible combinations of three compound classes requires substantial samples and resources. A realistic strategy may be to first establish efficacy of single compounds, then gradually explore the most promising combination regimens.

### 5.5 Cross-study comparison and evidence synthesis

Comparison across included studies reveals both consistencies and discrepancies in reported findings that warrant careful interpretation.

Glutamate metabolism: Animal studies uniformly report elevated retinal glutamate levels in diabetic models (range: 1.5-3.2 fold increase vs. controls), with the magnitude of elevation generally correlating with disease duration. However, the temporal relationship between glutamate accumulation and neuronal damage remains contested. Dionysopoulou et al. observed microglial activation preceding glutamate elevation, whereas Shivashankar et al. reported glutamate accumulation as the triggering factor. This discrepancy suggests potential model-dependent or stage-specific differences and highlights that glutamate dysregulation may be both cause and consequence of DR pathology.

- Evidence consistency rating: MODERATE — Animal studies show consistent direction of change but temporal relationships remain unclear.
- Arginine-NO metabolism: Findings are more heterogeneous. While most studies (8/11) report decreased eNOS activity in diabetic retinas, the direction of change in iNOS expression varies across models and time points, potentially reflecting differences in disease stage or inflammatory status at the time of measurement. This heterogeneity limits the ability to draw firm conclusions about optimal intervention strategies.
- Evidence consistency rating: LOW — Inconsistent findings across iNOS expression and intervention outcomes.
- Human metabolomic data: The few available human studies show moderate consistency, with elevated kynurenine and decreased arginine being the most reproducible findings across three independent cohorts. However, the correlation between systemic (plasma) and local (vitreous/retinal) metabolite levels has not been systematically evaluated, limiting the utility of peripheral biomarkers as surrogates for retinal metabolism.
- Evidence consistency rating: MODERATE — Consistent direction but limited sample sizes and lack of retinal-specific data.

## 6 Clinical translation: challenges and strategies

Despite demonstrating multi-target regulatory advantages in treating diabetic retinopathy (DR) through modulation of amino acid metabolism, natural compounds face multiple barriers in transitioning from basic research to clinical application. These challenges encompass pharmacokinetic limitations, drug delivery system design, quality control standardization, and drug-drug interactions. This section systematically analyzes these critical challenges and proposes corresponding solutions.

### 6.1 Pharmacokinetic and drug delivery bottlenecks

Natural compounds commonly face extremely low oral bioavailability, severely limiting clinical efficacy realization. Polyphenolic compounds such as resveratrol and quercetin typically exhibit oral bioavailability below 5%, primarily due to extensive intestinal metabolism and microbial degradation (141). While *in vitro* studies demonstrate effective concentrations of 10-50 μM for these compounds, post-oral administration plasma peak concentrations typically remain <0.5 μM, representing a nearly 100-fold discrepancy (142). The situation proves more severe for alkaloid compounds, with berberine demonstrating oral bioavailability of merely 0.5-2%, primarily limited by P-glycoprotein-mediated efflux and hepatic first-pass metabolism (126). This results in animal experimental doses of 150–200 mg/kg (translating to approximately 900–1200 mg/day in humans) yielding only 9–24 mg of active ingredient entering circulation when accounting for extremely low bioavailability, far below effective concentrations identified in *in vitro* studies. Curcumin presents similarly, with approximately 90% undergoing rapid hepatic metabolism to glucuronidated and sulfated conjugates post-oral administration (143).

Ocular delivery faces stringent limitations imposed by the blood-retinal barrier (BRB). The BRB, constituted by tight junctions of retinal capillary endothelial cells, demonstrates permeability <1% for most natural compounds (144). Pharmacokinetic studies of ginsenoside Rg1 reveal that retinal tissue concentrations following oral administration reach only 8-12% of plasma concentrations (140). Addressing these challenges, nanoformulation technology demonstrates potential. Li et al. (38) reported that curcumin lipid nanoparticles increased ocular drug concentrations 3-5-fold while extending intraocular retention time (145). Nanocarriers incorporating specific ligands can actively target retinal vascular endothelial cells, enhancing drug enrichment (146). However, these advanced delivery systems predominantly remain confined to animal experimental stages, with clinical translation progressing slowly, primarily constrained by complex manufacturing processes, high costs, and insufficient long-term safety data.

Comprehensive ADMET (Absorption, Distribution, Metabolism, Excretion, Toxicity) characteristic evaluation reveals additional challenges. Polyphenolic compounds undergo metabolism primarily via CYP2C9 and CYP3A4, potentially interacting with drugs metabolized through these enzymes (such as warfarin and glibenclamide) (147). Terpenoid compounds demonstrate plasma protein binding rates generally exceeding 95%, limiting free drug distribution to tissues (148). In DR patients, the high prevalence of diabetic nephropathy (approximately 30-40%) necessitates particular attention to drug accumulation risks under impaired renal function. Berberine clearance significantly decreases under impaired renal function, potentially increasing hypoglycemia and lactic acidosis risks (149). Existing clinical trials maintain follow-up periods of only 3–6 months (150–152), insufficient for evaluating chronic cumulative toxicity, with long-term safety data deficiency representing a major shortcoming in clinical application.

### 6.2 Quality control and standardization system construction

Natural plant-derived compound quality experiences influence from multiple factors including cultivation environment (soil, climate), harvest timing (season, growth cycle), and processing methods (drying, extraction, purification), leading to significant variations in active ingredient content and proportions (153). Resveratrol content in grapes from different origins can differ more than 10-fold, with this quality instability directly impacting clinical efficacy reproducibility. Beyond major active ingredients, plant extracts contain numerous minor components and impurities that may influence major component absorption, metabolism, or generate independent pharmacological effects.

Although multiple optimized extraction and purification methods have been proposed (154), these methods require specialized equipment and technical expertise while lacking standardized operational protocols. Substantial differences in extraction conditions employed by different research institutions render research results difficult to compare. Natural compound quality standardization currently remains in early stages, lacking unified standards and detection methods (153). Establishing comprehensive quality standard systems requires selecting active ingredients closely related to efficacy as quality control indicators. For compound formulations, simultaneous control of multiple major component contents and their ratios necessitates first clarifying independent effects and synergistic effects of each component through pharmacodynamic studies.

Analytical method development and validation require adoption of high-performance liquid chromatography (HPLC) and liquid chromatography-mass spectrometry (LC-MS) technologies (155). For complex natural extracts, chemical fingerprinting technology enables more comprehensive product quality consistency evaluation by comparing relative intensities and distribution patterns of multiple characteristic peaks (156). Production processes must comply with Good Manufacturing Practice (GMP) regulations, though natural ingredient complexity renders relevant standard and quality control procedure establishment more challenging. While multi-component combination may generate synergistic effects, it also increases quality control difficulty, with potential chemical reactions between components (such as oxidation-reduction, complexation) potentially reducing activity or generating new compounds (157). Sun et al. (158) indicate that insufficient understanding of inter-component interactions may affect efficacy and safety (158), necessitating comprehensive scientific evaluation of compound formulation development, including *in vitro* compatibility experiments, pharmacodynamic studies, and long-term stability testing.

### 6.3 Drug-drug interactions and clinical safety assessment

DR patients typically simultaneously employ multiple medications controlling blood glucose, blood pressure, and blood lipids, with natural compound interactions with these medications representing important clinical application safety considerations. Berberine possesses independent hypoglycemic effects through AMPK activation (121), potentially generating additive or synergistic effects when combined with metformin, theoretically enhancing hypoglycemic efficacy while potentially increasing hypoglycemia and lactic acidosis risks (159). Clinical studies demonstrate that berberine (1.5 g/day) combined with metformin achieves additional HbA1c reduction of 0.5-0.8%, though hypoglycemic event incidence increases from 8% to 15% (127). Quercetin may delay carbohydrate absorption through α-glucosidase inhibition, theoretically enhancing effects of medications such as acarbose (160).

Many DR patients with concurrent dyslipidemia require statin therapy. Berberine represents a moderate CYP3A4 inhibitor, potentially slowing clearance of CYP3A4-metabolized statins such as simvastatin and atorvastatin, increasing myopathy risk. Pharmacokinetic studies demonstrate berberine (300 mg three times daily) increases simvastatin plasma concentrations approximately 40% (131). Both polyphenolic compounds and berberine demonstrate certain antihypertensive effects, with resveratrol exhibiting vasodilatory effects through eNOS activation increasing NO production (161), theoretically potentially generating additive effects when combined with ACEI or ARB antihypertensive medications, though existing clinical trials report no significant hypotensive events (149, 152).

Polyphenolic compounds demonstrate platelet aggregation inhibitory effects in *in vitro* experiments (162). Quercetin functions as a CYP2C9 inhibitor, while warfarin undergoes metabolism primarily through CYP2C9, theoretically potentially slowing warfarin metabolism, enhancing anticoagulant effects and prolonging INR (147). For patients using aspirin or clopidogrel, the degree of increased bleeding risk with polyphenolic compound combination remains unclear. Other interactions requiring attention include: high-dose resveratrol may inhibit thyroid peroxidase activity, theoretically potentially affecting levothyroxine efficacy (163); curcumin functions as a P-glycoprotein(P-gp) and multiple CYP enzyme modulator, potentially affecting plasma concentrations of immunosuppressants such as tacrolimus and cyclosporine (164); some studies demonstrate polyphenolic compounds may interfere with pro-oxidative cytotoxic effects of certain chemotherapeutic drugs through antioxidant effects (165).

Existing clinical trial data demonstrate generally favorable short-term safety of natural compounds. Pooled data from three RCTs reveal overall adverse event incidence of 10-20%, predominantly mild gastrointestinal reactions (nausea 13.3%, diarrhea 7.8%, abdominal discomfort 4.4%), with no withdrawals due to adverse reactions and no serious adverse events (151, 152). However, long-term safety data deficiency represents a major shortcoming, with existing studies maintaining follow-up periods of only 3–6 months. For chronic disease treatment requiring 2–5 years or longer safety data verification. Most adverse events demonstrate dose-dependence, with blind dosage escalation potentially increasing toxicity risks. Pregnant women, lactating women, children, elderly patients, and those with hepatic or renal impairment demonstrate even greater safety data deficiency, necessitating cautious use or avoidance in these populations.

### 6.4 Combination therapy optimization and clinical translation strategies

Natural compound combination with conventional hypoglycemic medications can generate synergistic effects through different action mechanisms. Both berberine and metformin function through AMPK activation, though with differing action targets: metformin primarily inhibits hepatic gluconeogenesis, while berberine additionally improves insulin resistance through gut microbiota modulation (130). Clinical studies demonstrate berberine (1.0 g/day) combined with metformin achieving additional 0.5% HbA1c reduction after 3 months of treatment (166), with this synergistic effect potentially permitting metformin dose reduction while decreasing gastrointestinal adverse reactions. Polyphenolic compounds may protect pancreatic β-cell function through oxidative stress and inflammation improvement, with quercetin + resveratrol combined with standard hypoglycemic treatment achieving additional 0.7% HbA1c reduction (150). For insulin-requiring patients, animal experiments demonstrate resveratrol can reduce insulin requirements by 20-30% (104).

Based on systematic amino acid metabolism analysis, different natural compound classes target different metabolic pathways, with combination potentially achieving more comprehensive pathological intervention. Polyphenols primarily regulate glutamate metabolism and direct antioxidation, while alkaloids primarily target arginine-NO pathways. Animal experiments demonstrate resveratrol + berberine combination group pathological improvement (45%) significantly exceeding either single-use group (32-35%). Mechanism studies suggest resveratrol-reduced oxidative stress protects eNOS activity, enhancing berberine-promoted NO generation, while berberine-improved microcirculation promotes resveratrol delivery to retinal tissues. Polyphenol antioxidant effects and terpenoid anti-inflammatory effects demonstrate high complementarity, with resveratrol + ginsenoside Rg1 combination group ROS reduction (52%) and inflammatory factor reduction (58%) both significantly exceeding single-use groups, with lesion scores decreasing 55%. Cross-pathway analysis reveals oxidative stress reduction decreases inflammatory factor activation of IDO, while inflammatory factor reduction decreases ROS generation stimulation, forming positive synergistic cycles.

Combination therapy dose optimization requires balancing efficacy and safety. Fixed-dose ratio formulations facilitate patient use though cannot adjust according to individual differences, while individualized dose schemes offer greater flexibility at the cost of increased prescription complexity. Timing considerations necessitate attention to individual component characteristics: polyphenolic compounds experience significant food effects, with post-meal administration reducing gastrointestinal irritation though potentially decreasing absorption; berberine pre-meal administration may better exert carbohydrate absorption-delaying effects. Initiating at low doses with gradual escalation enhances safety and tolerability, with recommended initial doses of 50% target doses, gradually increasing to target doses over 2–4 weeks based on efficacy and tolerability. Despite preliminary evidence from animal experiments and small-sample clinical studies, rigorous prospective RCTs remain necessary for efficacy and safety confirmation. Based on primary outcome indicators (DR progression rates), ≥200 cases/group achieve adequate statistical power, with follow-up periods of at least 2 years evaluating DR progression delay long-term effects, including single-agent groups, combination groups, and placebo control groups employing factorial design analyzing independent effects and interactive effects of each component (167).

### 6.5 Frontier technologies and future development directions

Deep integration of metabolomics and network pharmacology constitutes an efficient paradigm for natural drug discovery. Metabolomics employing LC-MS platforms analyzes metabolic fingerprints of complex extracts, correlating “chemical composition-biological effects” on global scales, facilitating precise identification of small molecules strongly correlated with specific phenotypes from massive candidate pools (168). Network pharmacology employs “compound-target-pathway-disease” multidimensional networks systematically characterizing multi-target synergy and key pathways (169). Combined with high-throughput screening (HTS), researchers can parallelly evaluate thousands of compounds under automated conditions, substantially shortening timelines from “crude extracts” to “lead compounds” (170). Microfluidic chips provide novel HTS platforms, offering low material consumption, high sensitivity, and high reproducibility advantages (171). Integrating machine learning and computer-aided drug design (CADD) predicting activity probability, ADMET characteristics, and molecular interactions enables early elimination of low-value compounds, with bioinformatics and data mining integrating screening results with omics data and disease knowledge bases achieving traceable mapping of “candidate compounds-potential targets-key pathways” (172).

Addressing natural compound low bioavailability and ocular delivery barriers, nano-delivery technology demonstrates tremendous potential. Liu et al. (173) propose combining polyphenolic compounds with polysaccharides forming nanoplatforms can improve solubility and stability, enhancing efficacy while reducing adverse effects (173). Solid lipid nanoparticles and nanostructured lipid carriers protect lipophilic natural compounds from degradation through encapsulation, prolonging circulation time and improving tissue distribution (145). PLGA biodegradable polymer nanoparticles achieve sustained-release administration, reducing dosing frequency. Amphiphilic lipid or block copolymer self-assembled nanostructures can simultaneously load hydrophilic and lipophilic components, suiting multi-component compound formulation delivery. Cell-derived exosomes as natural nanocarriers demonstrate favorable biocompatibility and low immunogenicity, efficiently crossing biological barriers (174). However, these advanced delivery systems predominantly remain at laboratory research stages, with clinical translation facing complex manufacturing processes, high costs, quality control difficulties, and unknown long-term safety challenges, with regulatory agencies imposing more stringent approval requirements for nanoformulations (175).

Genotype and phenotype-based population stratification promises enhanced precision and efficacy of natural compound DR treatment. Genetic polymorphisms may influence efficacy and safety: NOS3 gene polymorphisms affecting eNOS activity may modulate berberine-promoted NO generation effects (176), MTHFR gene polymorphisms affecting homocysteine metabolism and oxidative stress may influence polyphenolic compound antioxidant effects (177), IDO1 gene polymorphisms may affect terpenoid compound tryptophan-kynurenine pathway regulation capacity. Baseline amino acid metabolic states may predict treatment responses: patients with high baseline glutamate levels may benefit more from polyphenolic treatment, while patients with high baseline Kyn/Trp ratios may respond better to terpenoid compounds. Gut microbiota composition influences natural compound metabolism and bioactivity, with Zhang et al. (130) discovering patients with specific microbiota compositions responding better to berberine treatment (130), with gut microbiota analysis potentially becoming treatment response predictive biomarkers while providing novel approaches for improving efficacy through probiotics or fecal microbiota transplantation.

Natural compound clinical translation requires adaptive regulatory frameworks and policy support. Traditional drug approval pathways may not fully apply to natural compound compound formulations, with US FDA botanical drug guidance and China NMPA traditional Chinese medicine new drug approval pathways providing alternative frameworks (178). Large electronic health record databases and real-world studies can supplement RCT evidence deficiencies, particularly in evaluating long-term safety and efficacy across different populations (179). Due to limited commercial incentives, governmental and philanthropic foundation funding proves critical for natural compound research, with NIH’s National Center for Complementary and Integrative Health specifically supporting natural product research (180), with strengthened international collaboration sharing research resources and data accelerating global natural compound development progress.

## 7 Conclusions

Natural compounds demonstrate unique multi-target regulatory advantages in DR treatment, though transitioning from laboratory to clinical application continues facing pharmacokinetic limitations, ocular delivery barriers, quality standardization, and drug-drug interaction challenges. Overcoming these challenges necessitates multidisciplinary collaboration, integrating latest advances in pharmaceutical chemistry, pharmaceutics, pharmacology, clinical medicine, and regulatory science. Nano-delivery technology, advanced screening methods, and precision medicine strategies provide novel tools for clinical translation, with establishing rigorous quality standard systems and comprehensive drug interaction databases constituting foundations ensuring safety and efficacy consistency. Future research should focus on: (1) developing high-bioavailability novel formulations, particularly ocular-targeted delivery systems; (2) establishing quality standards and detection methods for natural compound compound formulations; (3) systematically evaluating interactions with common diabetic medications and combination therapy schemes; (4) exploring population stratification strategies based on genotype, metabolic phenotype, and microbiota phenotype; (5) conducting multicenter, large-sample, long-term follow-up confirmatory clinical trials. Only through establishment on foundations of sufficient clinical evidence and rigorous quality control can natural compounds truly translate into effective DR treatment tools, providing patients more treatment options and improved prognoses.

Limitations and evidence gaps: It should be acknowledged that the current evidence base for natural compounds in DR treatment has significant limitations. The majority of mechanistic insights derive from animal models that may not fully recapitulate human disease pathophysiology. Human clinical data are scarce, with most studies involving small sample sizes (n < 50), short durations (< 12 weeks), and surrogate rather than clinical endpoints. The heterogeneity of study designs, outcome measures, and intervention protocols precludes quantitative meta-analysis. Therefore, while preclinical evidence suggests potential therapeutic benefits, current clinical evidence is insufficient to support therapeutic recommendations for any natural compound in DR management. Translation of these findings to clinical practice requires adequately powered, long-term randomized controlled trials with validated ophthalmological endpoints.

## Glossary

DR: Diabetic Retinopathy
NPDR: Non-Proliferative Diabetic Retinopathy
PDR: Proliferative Diabetic Retinopathy
RND: Retinal neurodegenerative changes
T2DM: Type 2 Diabetes Mellitus
ROS: Reactive Oxygen Species
TNF-α: Tumor Necrosis Factor-alpha
IL-6: Interleukin-6
CLDI: chronic low-grade inflammation
BCAAs: Branched-chain amino acids
NMDA: N-methyl-D-aspartate
IDO: indoleamine-2,3-dioxygenase
TDO: tryptophan-2,3-dioxygenase
NO: nitric oxide
eNOS: endothelial nitric oxide synthase
iNOS: inducible nitric oxide synthase
KP: kynurenine pathway
HTS: high-throughput screening
CADD: computer-aided drug design
ADMET: Absorption, Distribution, Metabolism, Excretion, Toxicity
AMPK: AMP-activated protein kinase
TCM: Traditional Chinese Medicine
